# Supplementary material for: A decision-space model explains context-specific decision-making
Source: Nat Commun. 2025 Aug 14;16:7437. doi: 10.1038/s41467-025-61466-x (PMC12354707; doi:10.1038/s41467-025-61466-x)
Supplement: Supplementary file 1 — Supplementary Information [file 41467_2025_61466_MOESM1_ESM.pdf]

Supplementary Information

A decision-space model explains context-specific decision-making

Beck, Dirk W.[1]; Heaton, Cory N.[2]; Davila, Luis D.[1#]; Rakocevic, Lara I.[1#]; Drammis, Sabrina M.[3#]; Tyulmankov, Danil[4#]; Giri, Atanu[1]; Umashankar Beck, Shreeya[2]; Zhang, Qingyang[5]; Pokojovy, Michael[6]; Negishi, Kenichiro[7]; Salcido, Alexis A.[2]; Reyes, Neftali F.[2]; Macias, Andrea Y.[2]; Batson, Serina A.[2]; Vara, Paulina[2]; Ibáñez Alcalá, Raquel J.[2]; Hossain, Safa B.[2]; Waller, Graham L.[2]; O'Dell, Laura E.[8]; Moschak, Travis M.[2]; Goosens, Ki A.*[9,10]; Friedman, Alexander*[1,2]

1. Computational Science Program, University of Texas at El Paso, El Paso, TX, USA
2. Department of Biological Sciences, University of Texas at El Paso, El Paso, TX, USA
3. Artificial Intelligence Laboratory, Department of Computer Science, Massachusetts Institute of Technology, Cambridge, MA, USA
4. Ming Hsieh Department of Electrical and Computer Engineering, Viterbi School of Engineering, University of Southern California, Los Angeles, CA, USA
5. Department of Biomedical Informatics, Harvard Medical School, Cambridge, MA, USA
6. Department of Mathematics and Statistics, Old Dominion University, Norfolk, VA, USA
7. National Institute on Drug Abuse, Baltimore, MD, USA
8. Department of Psychology, University of Texas at El Paso, El Paso, TX, USA
9. Department of Psychiatry, Icahn School of Medicine at Mount Sinai, New York, NY, United States
10. Center for Translational Medicine and Pharmacology, Icahn School of Medicine at Mount Sinai, New York, NY, United States

# Denotes 3rd authors who have contributed equally to the manuscript

*. Corresponding authors, please address ki.goosens@mssm.edu and afriedman@utep.edu.

Contents

Contents 2
Supplementary Figures 4
Supplementary Fig. 1 4
Supplementary Fig. 2 6
Supplementary Fig. 3 11
Supplementary Fig. 4 15
Supplementary Fig. 5 18
Supplementary Fig. 6 21
Supplementary Fig. 7 22
Supplementary Fig. 8 26
Supplementary Fig. 9 28
Supplementary Fig. 10 30
Supplementary Tables 32
Supplementary Table 1 32
Supplementary Table 2 34
Supplementary Table 3 37
Supplementary Table 4 44
Supplementary Table 5 46
Supplementary Table 6 48
Supplementary Table 7 51
Supplementary Notes 53
Supplementary Note 1 53
Supplementary Note 2 54
Supplementary Note 3 55
Supplementary Note 4 56
Supplementary Note 5 56
Supplementary Note 6 57
Supplementary Note 7 58
Supplementary Note 8 59
Supplementary Note 9 60
Supplementary Note 10 60
Supplementary Note 11 61

Supplementary Note 12 62
Instructions to run code. 63
Key resources table. 65
Supplemental References 67

Supplementary Figures

Supplementary Fig. 1: The model outputs the decision-space, action values, and choice based on the relative activities of circuit elements, Related to Fig. 1.

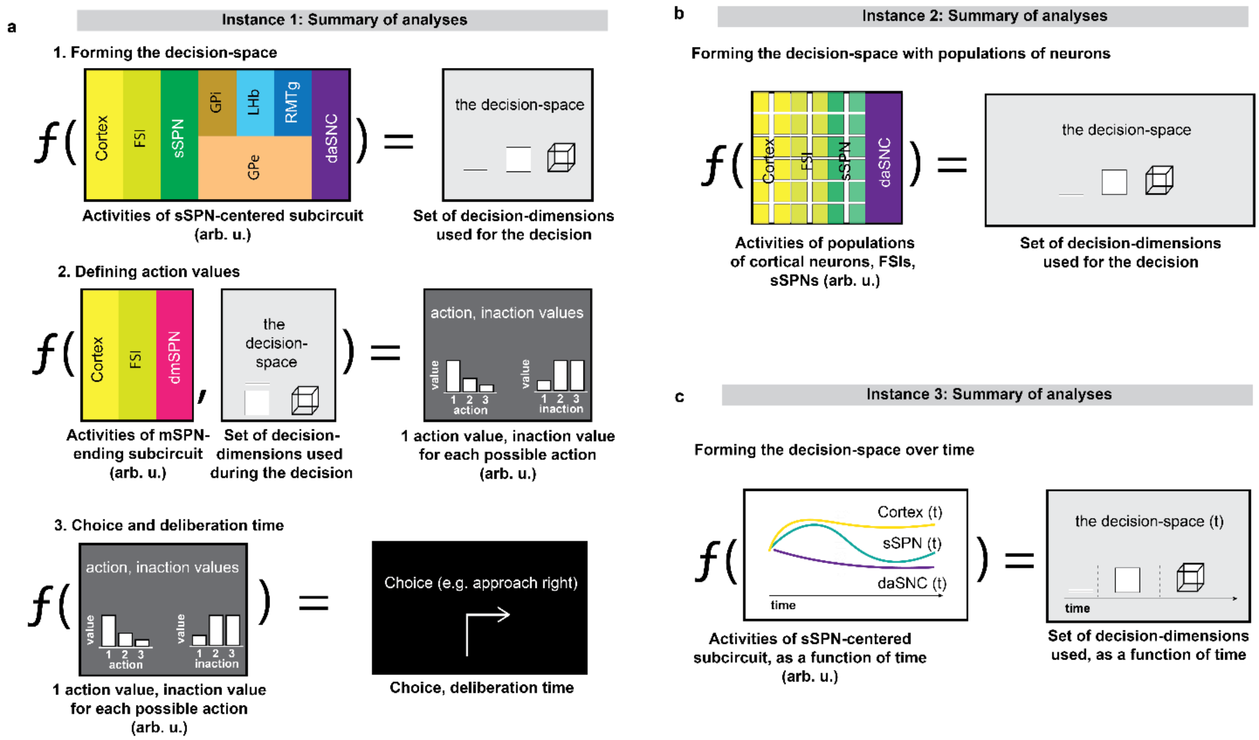

a, Primarily three types of analysis are performed using Instance 1 of the model (see **Fig. 1c., Instance 1: full connectivity and feedforward, Methods**). **1 (top row):** First, the decision-space is modeled based on the relative activities of the cortex, FSI, sSPN, GPi, LHb, RMTg, GPe, and/or daSNC. These analyses are plotted in, for example, **Figs. 2c-f, 3a,c 4f,g, 5b,c, 6a, 7, Supplementary Figs. 3p,q,s,u,w,x, 7a,b,g,h, 9a-c.** Note that here, activities are relative, and analysis is conducted in arbitrary units. This allows for directional effects of activities (e.g. sSPN activation leads to a lower-dimensional decision-space) to be modeled without fitting firing rates directly, allowing us to test our model against experimental data that records a portion, but not all, of the brain regions we model. There are many total parameters across the brain regions, but for each analysis, we set all but several parameters to a default value (see **Common parameters, Rationale behind parameter choices, Methods**). **2 (middle row):** Second, in some analyses, action values are modeled based on the relative activities of the cortex, FSI, and/or mSPN, and the decision-space. These analyses are plotted, for example, in **Figs. 3d, 4c-e,h, 7b,c,e, Supplementary Figs. 3q, 7c,d, 8d,f. 3 (bottom row):** Third, in some analyses, choice and/or

deliberation time is modeled, given action values. These analyses are plotted, for example, in **Fig. 3b, 4b, Supplementary Figs. 3c, 6, 7f,i.** For a proof of concept, we show that the model roughly reproduces the two-arm T-maze task in **Supplementary Fig. 3c.** Elsewhere, our analysis focuses on relative changes in choice and/or deliberation time (e.g. increasing the action value of approaching increases approach rate and decreases deliberation time).

b, Our analyses using Instance 2 of the model focus on the encoding of the decision-space by populations of cortical neurons, FSIs, and sSPNs (see **Supplementary Fig. 5, Instance 2: sparse connectivity and feedforward, Methods**).

c, Instance 3 of the model is used to analyze how the decision-space is formed over time via the interactions between the cortex, sSPN, daSNC, and mSPN (see **Fig. 5, Supplementary Fig. 7, Instance 3: full connectivity and dynamics**).

Supplementary Fig. 2: Action values are defined within a decision-space formed by the circuit, Related to Figs. 1,2.

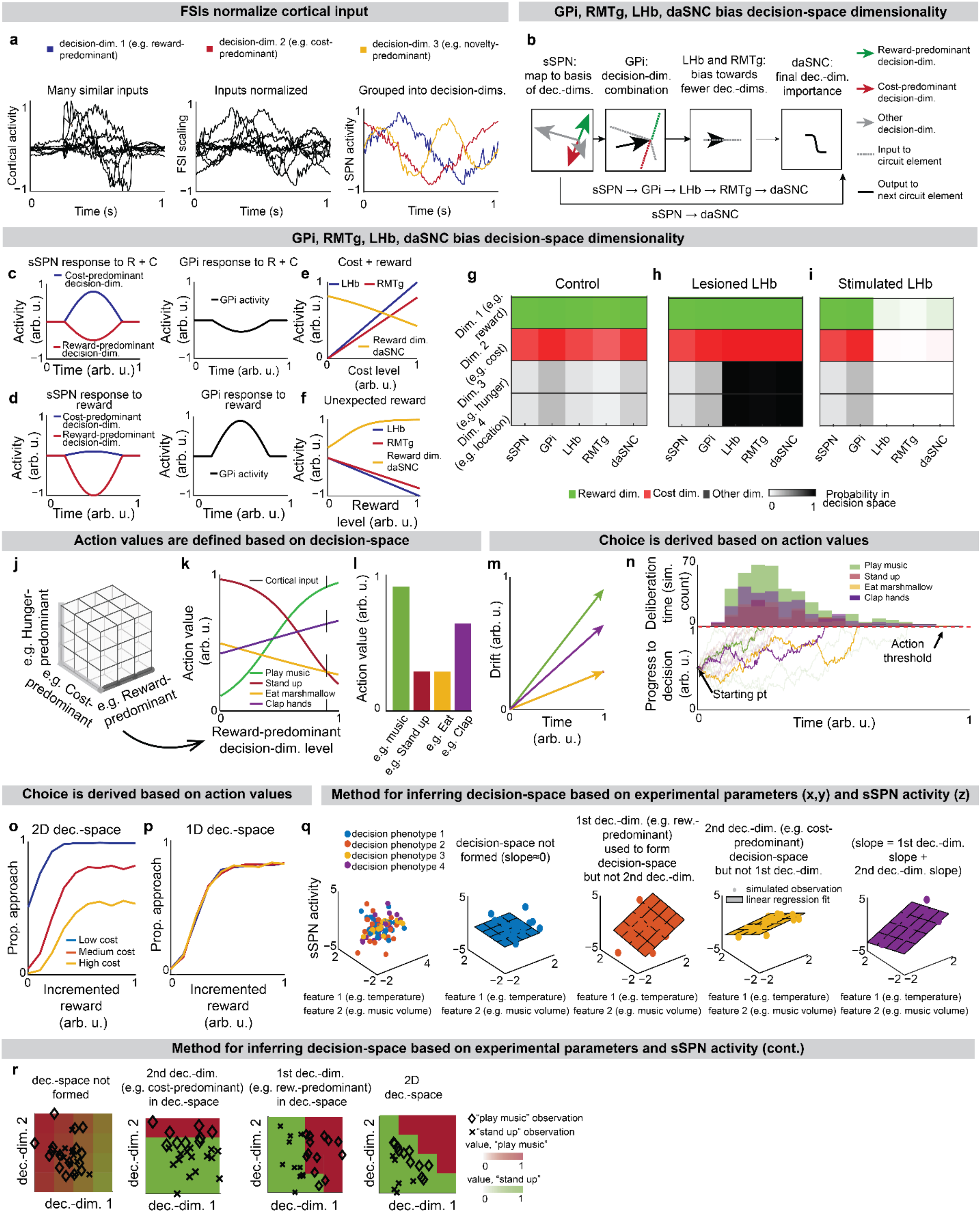

a, Example showing how FSIs and SPNs parse cortical activity in the model. The signals of 10 cortical neurons encoding sensory information (**a**) are normalized by FSIs such that activities are on a more uniform scale (**b**) and then mapped to sSPNs (**c**), which each encode activity along a "decision-dimension." Here, Instance 1 of the model in **Fig. 1c** is used. Also see ***Instance 1: full connectivity and feedforward,*** **Methods**.

b, After FSI normalization, sSPNs map cortical activity to a basis of decision-dimensions. Mathematically, we represent, for a given pathway P (either direct pathway or indirect pathway), the mapping of cortical activity $\mathbf{x}_P$ to decision-dimensions as a linear transform via the matrix $\mathbf{W}_P$, whose columns correspond to the first several (in our analysis, 4) principal components of cortical activity. During this process, there is divisive normalization by FSI activity c_P and the potential for an overall shift in sSPN activity b_{sSPN} (eq. (1)). Next, GPi combines the sSPN signals into a single representation and RMTg and LHb bias this combined representation of signal along all decision-dimensions. We represent RMTg activity RMTg as the dot product of striosome to GPi weights $\mathbf{w}_{\mathrm{GPi}}$ and the activities the sSPNs $\mathbf{s}_{\mathrm{sSPN},P}$ for each pathway P, after incorporation of additive shifts z_{GPi}, z_{LHb}, and z_{RMTg} (eq. (6)). Next, daSNC takes input directly from sSPNs (in the direct pathway), from striosomes through GPe (in the indirect pathway), and from RMTg to calculate the final importance of each decision-dimension. We represent daSNC activity $\mathrm{daSNC}_{i,P}$ corresponding to decision-dimension i and pathway P as the activity of the corresponding sSPN population $\mathbf{s}_{\mathrm{sSPN},i,P}$, added to GPe activity in the indirect pathway ($z_{\mathrm{GPe},P}$, $z_{\mathrm{GPe,Direct}}=0$), multiplied by a connection weight $w_{\mathrm{sSPN}\rightarrow\mathrm{daSNC},i,P}$, plus additive shifts applied individual to each daSNC element ($z_{\mathrm{daSNC},i,P}$) and uniformly to all daSNC elements (RMTg), all passed through an activation function (eq. **(2)**).

c,d, Example where sSPNs parse reward and cost (**c**), or reward (**d**) inputs along decisiondimensions (left) and then GPi combines the signals along the decision-dimensions into a single representation (right).

e,f, Modeled responses of LHb, RMTg, and daSNC to a cortical signal encoding reward and cost (**e**), and a cortical signal encoding an unexpected reward (**f**). LHb and RMTg increase their activities proportionally to the cost and decrease their activities proportionally to the reward. daSNC change their activities inversely. The modeled daSNC response is due to the combination of cortical inputs projected onto them directly from sSPN and through GPi, LHb, and RMTg.

g-I, Roles of the circuit elements in determining which dimensions form decision-space. Three scenarios are shown: control ($z_{\text{LHb}}=0.5$ in eq. (6)), lesioned LHb ($z_{\text{LHb}}=-5$), and stimulated LHb ($z_{\text{LHb}}=5$). The colors shown for each circuit element correspond to the decision-space that would be formed absent the influence of downstream circuit elements.

j-l, Process by which action values are defined using decision-space. During a decision, a subset of decision-dimensions is selected, forming decision-space (**h**). This is represented mathematically through a diagonal matrix $\mathbf{S}_P$ whose elements are set probabilistically to either 1 (dimension in decision-space) or 0 (dimension not in decision-space) (eq. (3)). Otherwise, mSPN activity is formulated similarly to sSPN activity. Rules corresponding to retained decision-dimensions are used to define action values $v_{j,P}$ for action j and pathway P (**k,l**). This is represented mathematically as multiplication of a vector $\boldsymbol{\beta}_{j,P}$ by mSPN activity $\mathbf{s}_{\text{mSPN},P}$, subtracted by a shift $\alpha_{j,P}$ and run through an activation function (eq. (4)).

m,n, Action (or inaction) values j for each action j and pathway P are used as drift rates (**m**) in a Merton process model (**n**). Discrete Merton processes obtained as a constant time step discretization of eq. (8) are run for each action simultaneously. At the time the first of the $v_{j,\text{direct}}$ processes reaches a defined threshold h, the corresponding action is enacted unless its inaction process $v_{j,\text{indirect}}$ has reached h first (eqs. (9),(10),(11)). Lines show the progress of processes towards a decision threshold for an example simulation. Histogram shows the decisions and deliberation times for the processes that reached the threshold first.

o,p, Psychometric functions derived across multiple reward levels. In the modeled experiment, the subject is asked to evaluate the rewards and costs of two offers and either approach or avoid. The decision-space (**o**: 1D, **p**: 2D) affects choice patterns.

q,r, Demonstration of a method by which decision-space can be inferred from sSPN activity, showing the utility of the mathematical formation of the model in connecting experimental inputs, sSPN activity, and choice. The method demonstrated here could be used to design an experiment in which the decision-space model is tested, or it could be used to explain differences in choice between groups, for instance control and disorder. In the simulation, two environmental inputs (e.g. temperature, music volume) across 100 sessions are classified based on decision-making phenotypes, for example based on reaction time, heart rate, eye movement (colors). Using the method visualized here, the decision-making phenotype classes are assigned one of four

decision-space reference labels: 1) where decision-space is not formed, 2) where only the first decision-dimension is used to form decision-space, 3) where only the second decision-dimension is used to form decision-space, and 4) where both decision-dimensions are used to form decisionspace. Synthetic data is generated by forming an arbitrary set of cortex→sSPN weights, using these to form sSPN activity, and then adding i.i.d. Gaussian noise. A linear regression is used to derive estimated decision-dimensions and assign hypothesized decision-spaces to each label. Choice is then examined with respect to the derived decision-dimensions. As expected, the regression slope (planes) corresponding to the 2D decision-space is roughly the sum of the regression slopes of the two 1D decision-spaces (**q**), and decisions correlate with the dimensions hypothesized to be used to form decision-space when choices are plotted against hypothesized dimensions (**r**). In the colormaps in **r**, action values are interpolated from an example set of observations (diamonds and Xs) via logistic regression, and a boundary line is drawn where the action value of “play music” equals the action value of “stand up.”

(1) $$\mathbf{s}_{\mathrm{sSPN},P} = \frac{1}{c_P}\mathbf{W}_P{}^{\mathrm{T}}\mathbf{x}_P + b_{\mathrm{sSPN}}$$ (copied for convenience)

(2) $$\mathrm{daSNC}_{i,P} = \frac{1}{1 + \exp\left(w_{\mathrm{sSPN}\to\mathrm{daSNC},i,P} \cdot \left(\mathrm{s}_{\mathrm{sSPN},i,P} + z_{\mathrm{GPe},P} \right) + \mathrm{RMTg} - z_{\mathrm{daSNC},i,P} \right)}$$

(copied for convenience)

(3) $$\mathbf{s}_{\mathrm{mSPN},P} = \frac{1}{c_P}\ \mathbf{S}_P\mathbf{W}_P{}^{\mathrm{T}}\mathbf{x}_P$$ (copied for convenience)

(4) $$v_{j,P} = \frac{1}{1 + \exp\left(-\boldsymbol{\beta}_{j,P}\ \mathbf{s}_{\mathrm{mSPN},P} - \alpha_{j,P} \right)}$$ (copied for convenience)

(6) $$\mathrm{RMTg} = z_{\mathrm{RMTg}} + z_{\mathrm{LHb}} + z_{\mathrm{GPi}} \cdot \mathbf{w}_{\mathrm{GPi}} \cdot \begin{bmatrix} \mathbf{s}_{\mathrm{sSPN,\,direct}} \\ \mathbf{s}_{\mathrm{sSPN,\,indirect}} \end{bmatrix}$$ (copied for convenience)

(8) $$\mathrm{d}Y_{j,P} = v_{j,P}\,\mathrm{d}t + \sigma\,\mathrm{d}W_{j,P}\,,\ Y_{j,P}(t=0) = 0,\ \text{where } W_{j,P} \text{ is a standard Wiener process}$$

(copied for convenience)

(9) $$t_{\mathrm{action},j} = \min_t \left\{ t \mid Y_{j,\mathrm{direct}} \geq h \right\}$$

(10) $$t_{\mathrm{inaction},j} = \min_t \left\{ t \mid Y_{j,\mathrm{indirect}} \geq h \right\}$$

(11) $$\mathrm{action} = \arg\min_{j \in J} \left(Y_j(t_{\mathrm{action},j}) \right),\ \text{where } J \text{ is the subset of actions s.t. } t_{\mathrm{action},j} < t_{\mathrm{inaction},j}$$

Supplementary Fig. 3: Decision-space model validation and comparison to alternative models, Related to Fig. 3.

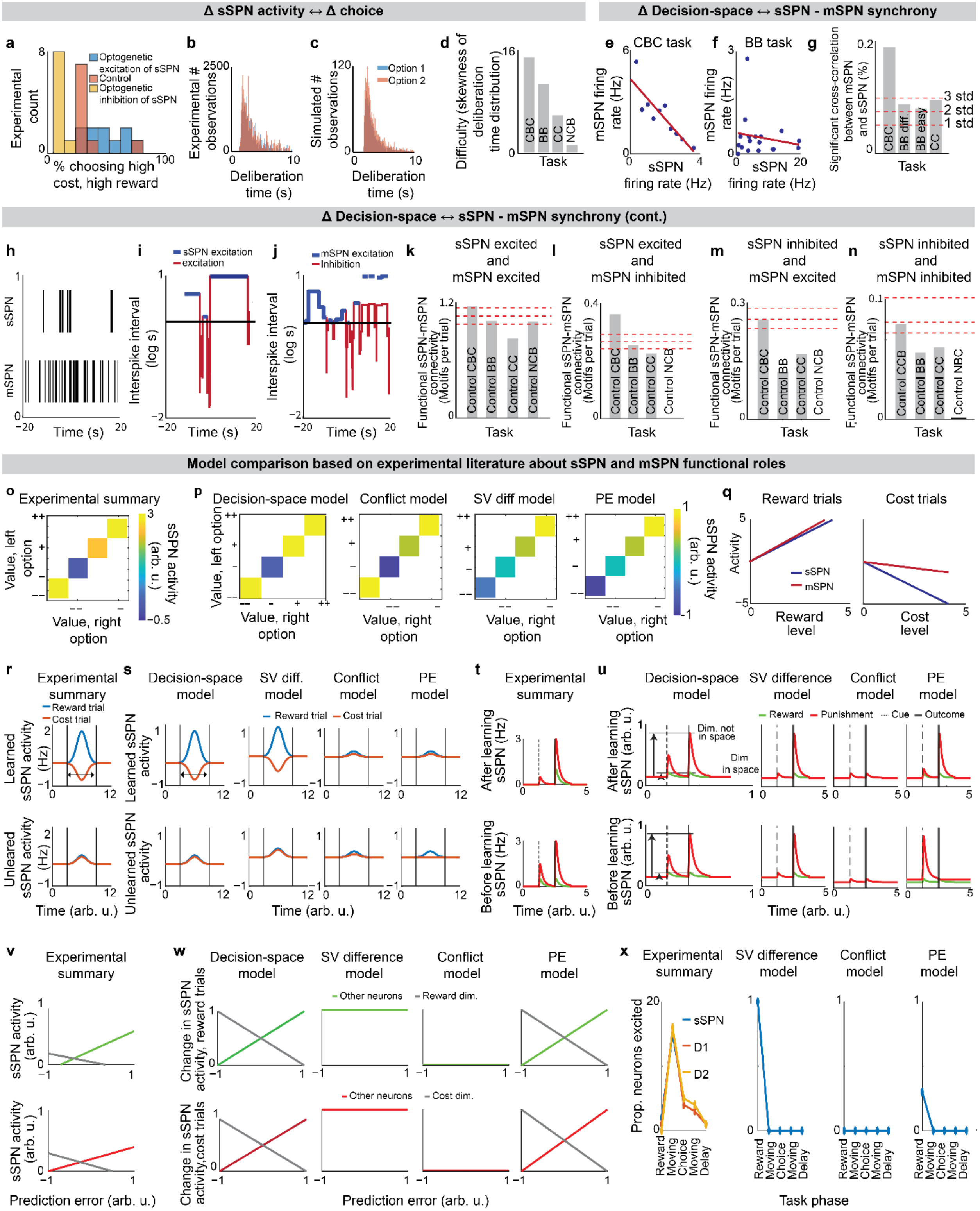

a, Summary of experimental results of optogenetic manipulation during a conflict task in Friedman et al. (2015). Resembles the model in **Fig. 3b**.

b,c, Experimental deliberation time distribution (6 animals, 35 sessions) (**b**), which is successfully modeled using the Merton process model (**c**). Distribution is for the benefit-benefit task (concentration 70%). Experimental data here and throughout the figure is analyzed from the Corticostriosomal Circuit Stress Experiment database. Source data are provided as a Source Data file.

d, Skewness of the deliberation time distribution (dimensionless units), which is used to estimate task difficulty. The CBC task had a deliberation time distribution that was more skewed than the non-conflict tasks. Tasks: NCB = non-conflict cost-benefit (4 rats, 27 sessions, 1250 trials), CC = cost-cost (7 rats, 25 sessions, 1852 trials), BB = benefit-benefit (7 rats, 128 sessions, 7762 trials), CBC = cost-benefit conflict (8 rats, 69 sessions, 3921 trials). Source data are provided as a Source Data file.

e,f, Analysis of relationship between sSPNs and mSPNs during decision-making. sSPN and mSPN neurons are significantly more correlated in tasks that require integration of reward and cost versus only reward or only cost. **e** shows representative examples from the cost-benefit conflict task (CBC, both reward and cost) and **f** shows the benefit-benefit task (BB, only reward), respectively. Source data are provided as a Source Data file.

g, The CBC task had significantly more correlated (Pearson's $r^2 > 0.4$ and significance < 0.05) pairs than the tasks which required integration of only reward or only cost. Confidence intervals (dashed red lines, 1,2,3 standard deviations) are estimated based on shuffled data. NCB = nonconflict cost-benefit (14 sSPNs, 260 mSPNs), CC = cost-cost (46 sSPNs, 400 mSPNs), CBC = cost-benefit conflict (84 sSPNs, 717 mSPNs), BB = benefit-benefit easy (50 sSPNs, 515 mSPNs, chocolate milk concentration <50), BB = benefit-benefit difficult (33 sSPNs, 731 mSPNs, chocolate milk concentration >=50). Source data are provided as a Source Data file.

h-j, Process by which we identify functionally connected sSPN and mSPN neurons. Spiking times (**h**) are used to find inter-spike intervals (**i**,**j**) for sSPN (top rows) and mSPN (bottom rows) that were recorded simultaneously during decision-making. Intervals above the median interval (black line) are classified as inhibition (blue) and below median are classified as excitation (red).

Functional connections are determined based on whether excitation or inhibition of sSPN followed by excitation or inhibition of mSPN. Source data are provided as a Source Data file.

k-n, Significantly more sSPN and mSPN neurons were functionally connected during decisions that required integration of reward and cost (CBC task) than the other tasks for all types of connections: sSPN excited and mSPN excited (**k**), sSPN excited and mSPN inhibited (**l**), sSPN inhibited and mSPN excited (**m**), and sSPN inhibited and mSPN inhibited (**n**). Tasks: NCB = nonconflict cost-benefit (14 sSPNs, 260 mSPNs), CC = cost-cost (46 sSPNs, 400 mSPNs), BB = benefit-benefit (83 sSPNs, 1246 mSPNs), CBC = cost-benefit conflict (84 sSPNs, 717 mSPNs). Source data are provided as a Source Data file.

o, Mean sSPN activity should track decision-space dimensionality. Tasks in Friedman et al. (2015), plotted in order of difference between reward and cost, are assessed for likely decisionspace dimensionality based on task difficulty (**Supplementary Fig. 4d**). Those with higher task difficulty are assigned lower sSPN activity per the model in **Fig. 3c**.

p, Three alternative models applied to the task in Friedman et al. (2015). The subjective value (SV) model assumes that sSPN encoded the relative action values of the two arms of the T-maze. The conflict model assumes that sSPN activity is inversely proportional to the amount of conflict in the task. The prediction error model compares expected value entering the task with reward or cost obtained on the maze. The conflict method resembles experimental results but not the SV difference model or the conflict model.

q,r, Summary of differences in sSPN activity across trials in the operant conditioning task in Friedman et al. (2020). mSPNs and sSPNs were active during reward trials, suggesting per the model in **Fig. 3c** that the decision-space was not formed (**q**). sSPNs were less active during the cost trials and were differently active than mSPNs, suggesting formation of a low-dimensional decision-space per the models in **Fig. 3c,d** (**r**)**.**

s, Prediction of sSPN activity for the task in **r** for the decision-space model and three alternative models. sSPN activity scales with overall subjective value in the task, so the subjective value model successfully interprets the experimental results.

t,u, Experimental results are summarized from Xiao et al. (2021) (**t**). Prediction of the decisionspace model and three alternative models of sSPN subpopulation activity at the cue and during the outcome period of a Pavlovian conditioning task (**u**). Per the decision-space model,

sSPN subpopulations should respond similarly to the cue and to the outcome because associated data is likely mapped along the same decision-dimension. The subjective value model expects more activity during the outcome period when reward is administered. The prediction error model expects more activity at the cue after learning. There was no conflict in the experimental setup.

v,w, Experimental results are summarized from Bloem et al. (2022) (**v**). Predictions of the decision-space model and the three alternative models of sSPN activity during a probabilistic Bandit task (**w**). The decision-space model expects that sSPN activity will reveal prediction errors, as does the prediction error model. The subjective value model instead anticipates that activity will track overall value regardless of prediction error. There was no conflict anticipated by conflict model.

x, In the value-guided choice task in Weglage et al. (2021), activities of all neuron types recorded (sSPN, dmSPN, imSPN) resembled one another over phases of the task and did not solely track subjective value, prediction error, or conflict. As shown in **Fig. 3d**, the experimental finding is an expectation of the decision-space model when a high-dimensional decision-space is formed.

Supplementary Fig. 4: Analysis of neural and decision-making data shows that decision-space is changed after stress, Related to Fig. 4.

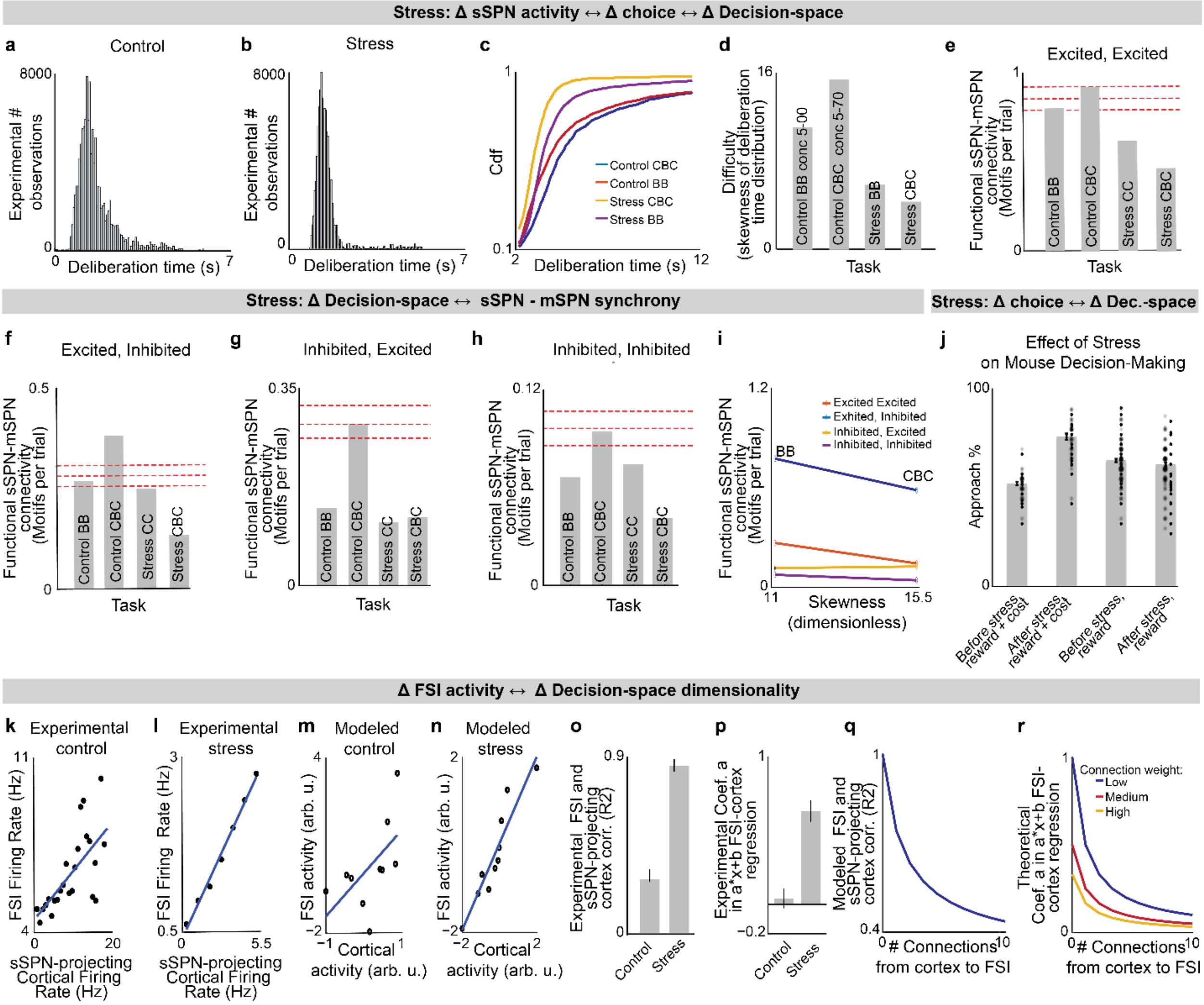

a,b, Deliberation time distributions of rodents performing the cost-benefit conflict task before stress (**a**) are less skewed and have shorter deliberation time than after stress (**b,** control: 8 rats, 198 sessions, 11683 cells; stress: 5 rats, 138 sessions). Source data are provided as a Source Data file.

c,d, Cumulative distribution functions (**c**) and distribution skewness (**d**) of deliberation time show that after chronic stress, the task involving integration of both reward and cost (CBC task) changes from producing the slowest (blue) to the quickest (yellow) decisions. CBC choice was slowest in control rats ($p < 0.0001$, KS-test). After stress, CBC choice was faster than in the other tasks

(p<0.0001). Control CBC task: 69 sessions, 8 rodents; Control BB task: 128 sessions, 7 rodents; Stress CBC task: 34 sessions, 5 rodents; Stress BB task: 104 sessions, 5 rodents. Source data are provided as a Source Data file.

e-h, sSPNs and mSPNs were more functionally connected during a difficult (CBC) task before stress than after, per all possible types of connection: sSPN excited, mSPN excited (**e**), sSPN excited, mSPN inhibited (**f**), sSPN inhibited, mSPN excited (**g**), and sSPN inhibited, mSPN inhibited (**h**). Significance levels, depicted by dashed lines, show one (bottom), two (middle), and three (top) STD for functional connections calculated from shuffled control data. Control costbenefit conflict (CBC) task: 92 pairs, 8 rodents; Tasks: Control BB = benefit-benefit (83 sSPNs, 1246 mSPNs), Control CBC = cost-benefit conflict (84 sSPNs, 717 mSPNs), Stress CBC = costbenefit conflict (41 sSPNs, 898 mSPNs), Stress BB = benefit-benefit (156 sSPNs, 2813 mSPNs). Source data are provided as a Source Data file.

i, Deliberation time distribution skewness is no longer linked to functional connectivity after stress. Source data are provided as a Source Data file.

j, Experimental data that inspires the model in **Fig. 4h**. Mice that underwent chronic stress approached the lower-reward arm of the T-maze less when a small cost was added ("reward + cost" = cost-benefit conflict task, “reward” = benefit-benefit task). Dots = individual sessions, bar = mean across trials. There is significant difference ($p<10^{-19}$, paired t-test) in choice for the CBC task before and after stress, and nonsignificant difference in choice for the BB task before and after stress (p=0.10). CBC, before stress: 17 rodents, 38 sessions; CBC, after stress: 13 rodents, 34 sessions; BB, before stress: 23 rodents, 114 sessions; BB, after stress: 14 rodents, 116 sessions. Our model interprets this result as due to differences in the decision-space. Source data are provided as a Source Data file.

k-n, Representative examples of simultaneously recorded FSIs and prelimbic cortex neurons firing rates before (**k**, Pearson’s R=0.46) and after (**l**, R=0.99) chronic stress. Source data are provided as a Source Data file. After stress, there is less coordination between the connected pairs. This can be modeled as a reduction in the number of cortical neurons that synapse to each FSI (**m,n**).

o-r, In general, the neuron pairs in rats that underwent chronic stress had significantly higher correlation (**o**, $p<10^{-18}$) and significantly higher values of slope “a” in their a.x+b linear regression

fits (**p**, $p<10^{-6}$). Control: 7 rodents, 78 neuron pairs; Stress: 4 rodents, 37 neuron pairs. Source data are provided as a Source Data file. This suggests, per our modeling, that there are fewer connections from cortical neurons to FSI after stress. Modeled squared Pearson correlation coefficient (**q**) and slope "a" parameter in the a.x+b fits (**r**) are shown when the connection between cortical and FSI neurons are altered in two ways: 1) through a reduction in the number of connections, and 2) through a reduction in the strength of each connection (i.e. connection weight). This experimental evidence is aligned with a reduction in the number of connections, suggesting that FSI normalization is disrupted after stress, leading to higher sSPN activity and thus formation of lower-dimensional decision-spaces.

Supplementary Fig. 5: Mean and variance of SPN activities reveals decision-space.

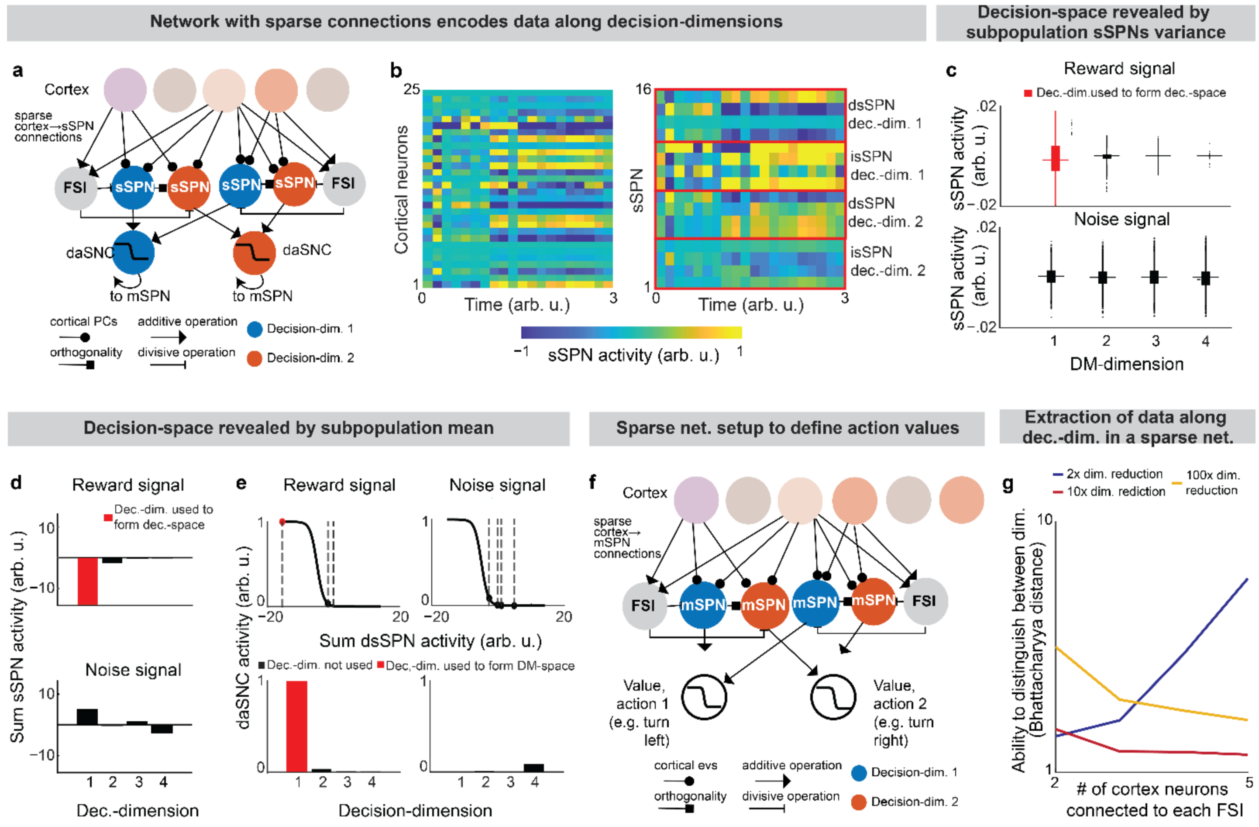

a,b, In this figure, we demonstrate that SPNs can encode activity along decision-dimensions successfully even in large, sparse networks, as exist in the brain, by considering an instance of the model where there are sparse connections between cortical neurons (n=50), FSIs (n=10,000), and SPNs (40,000 sSPNs, 40,000 mSPNs). sSPNs each encode activity along a principal component of a randomly sampled set of cortical neurons C (**a**). So, when activity changes in C (for example, during the reward cue in **b,** left panel), sSPNs (**b,** right panel) that each encode data along an ith principal component respond somewhat similarly to one another. See ***Instance 2: sparse connectivity and feedforward*****, Methods**.

c, Decision-space is revealed from the variance of sSPN activities, aligning to the experimental result in **Fig. 3e**. Modeled sSPN subpopulations have greater variance when there is highmagnitude cortical signal along a corresponding decision-dimension (e.g., an sSPN subpopulation corresponding to a reward-predominant decision-dimension in response to a reward cue). Summaries are shown of the activities of 10,000 dsSPN (top row). Two types of cortical signal are passed to SPNs: one with reward (left column, matches the example used in **Fig. 5**) and one with Gaussian white noise (right column). To produce the network, 10,000 groups

of 4 randomly sampled cortical neurons (notated as the set C) are connected to an FSI and 4 dsSPNs (or isSPNs) and 4 dmSPNs (or imSPNs) for each pathway. The activity of a given sSPN which receives projection from C is defined mathematically based on the weights from cortical neuron q to sSPN s, $w_{q \to s}$, the activity of the FSI which received projection from C, FSI_C, and an additive shift that represents the relative activity of all sSPN neurons, b_{sSPN} (eq. (22)).

d, Decision-space is revealed by the mean of sSPN activities, aligned with the model in **Fig. 3c**. An sSPN subpopulation has lower mean activity when there is high-magnitude cortical signal along a corresponding decision-dimension.

e, Selection process by which daSNC neurons determine which decision-dimensions to include and to not include in decision-space, illustrating the construction of decision-space from sSPN activity. Lines are daSNC activation functions. Placement along the x-axis (emphasized by dashed lines) is the subpopulation average activity in **d**. Mathematically, daSNC neuron corresponding to decision-dimension i and pathway P, $\mathrm{daSNC}_{i,P}$, is computed as the weighted average of sSPNs corresponding to that decision-dimension and pathway, shifted by RMTg activity RMTg and a daSNC biasing factor $z_{\mathrm{daSNC}, i, P}$, all passed through an activation function (eq. (23)).

f, Correlate to the sSPN-centered subnetwork described in **a** for mSPN, illustrating how action values could be defined by a network with sparse connections. The activity of a given mSPN which receives projection from C, $\mathrm{mSPN}_{m,C}$, is defined similarly to an sSPN but for term representing dopamine signaling from the daSNC neuron corresponding to decision-dimension i and pathway P to an mSPN corresponding to the same decision-dimension and pathway, $d_{i,P}$ (eq. (24)).

g, Bhattacharya distance between the distributions of SPNs encoding data along the rewardpredominant and cost-predominant decision-dimensions. sSPN can correctly differentiate reward from cost signal despite sparse cortico-striatal connectivity. Lines show the averages of 1000 simulations. This result demonstrates the feasibility of data along decision-dimensions being encoded by neural populations with sparse connections.

(22) $$\mathrm{sSPN}_{s,C} = \frac{1}{|C|} \sum_{q \in C} \frac{w_{q \to s}\, \mathrm{cortex}_q}{\mathrm{FSI}_C} + b_{\mathrm{sSPN}}$$

(23)
$$\mathrm{daSNC}_{i,P} = \frac{1}{1+\exp\left(\frac{1}{n_{\mathrm{sSPN}}}\sum_{s\in i,P} w_{s\to \mathrm{daSNC},i,P}\cdot \mathrm{sSPN}_s + \mathrm{RMTg} - z_{\mathrm{daSNC},i,P}\right)}$$

(24)
$$\mathrm{mSPN}_{m,C} = \frac{d_{i,P}}{|C|}\sum_{q\in C}\frac{w_{q\to m}\,\mathrm{cortex}_q}{\mathrm{FSI}_C}$$

Supplementary Fig. 6: The decision-space is differentially constructed based on cortical signalto-noise ratio (SNR).

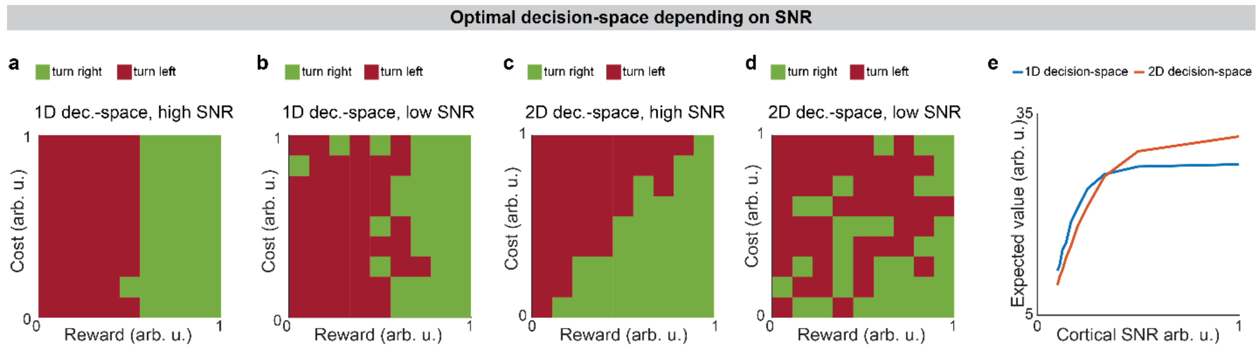

a-d, Modeled T-maze task where an animal turns right to choose a reward/cost offer or turns left to avoid it. Cases where there is high cortical SNR (**a**,**c**) or low cortical SNR (**b,d**) and a 1D decision-space (**a,b**) or 2D decision-space (**c,d**) are formed. Modeled "turn right" actions are considered successful (positive value) when reward > cost, and "turn left" when reward < cost. 2D decision-spaces lead to more value when there is low cortical SNR (**c**) but not when there is high SNR (**d**).

e. Choices using different types of decision-spaces have different expected reward minus cost (expected value), depending on cortical SNR.

Supplementary Fig. 7: Roles of the direct and indirect pathways, Related to Fig. 5.

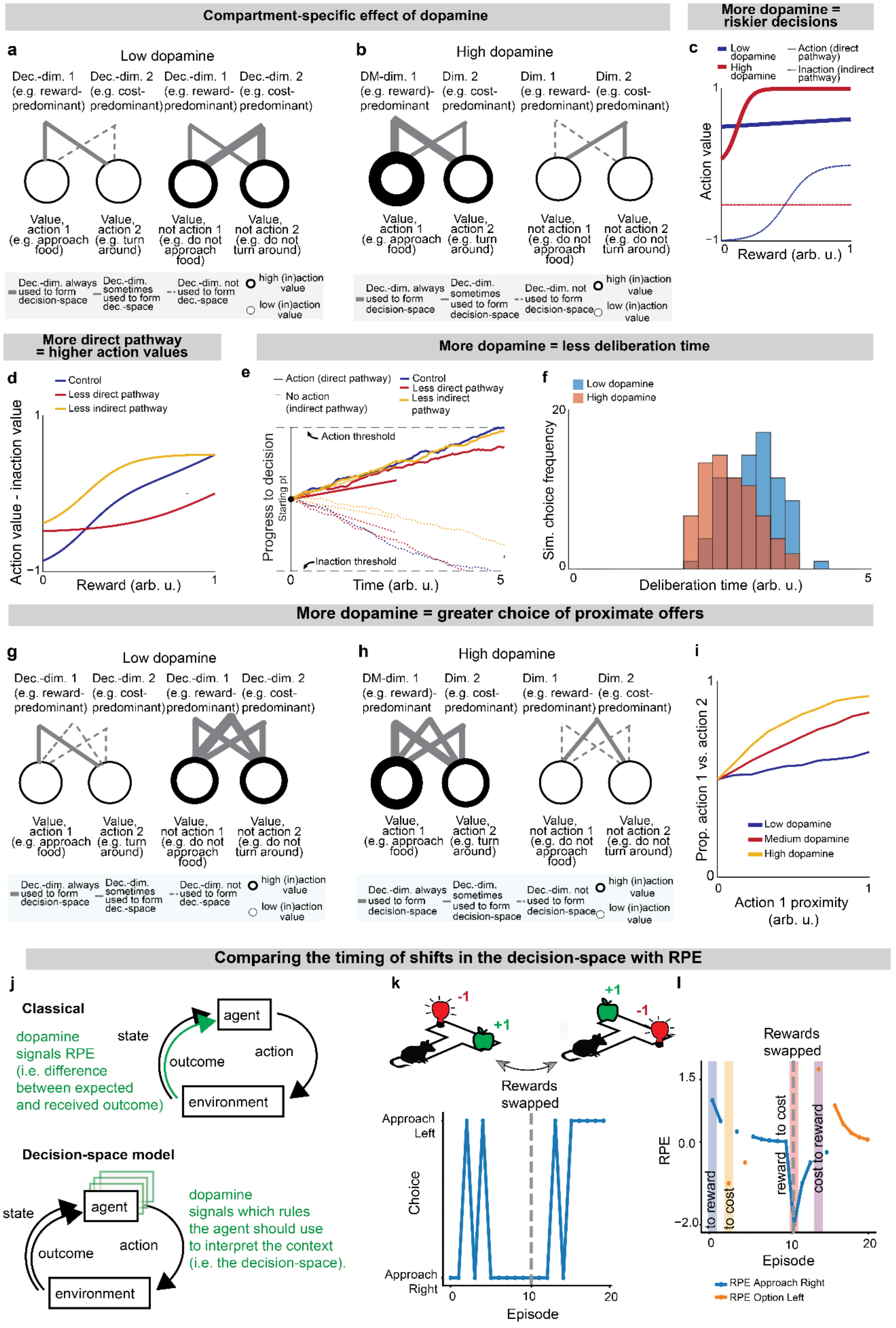

a,b, Effect of low (**a**) versus high (**b**) dopamine release on decision-space formed by the direct pathway (left panels) or indirect pathway (right panels). When dopamine release is low (**a**), lowdimensional direct pathway decision-spaces are constructed by dsSPNs and high-dimensional indirect pathway decision-spaces are constructed by isSPNs. The opposite happens when there is high dopamine (**b**). To analyze these effects, analysis in **Fig. 5** uses an instance of the model where the circuit elements interact dynamically, represented mathematically through a system of differential equations, where sSPN activity for a given decision-dimension i and pathway P, $s_{i,P}(t)$, respond to cortical input after normalization by FSI, $x_{i,P}(t)$, based on weights w between sSPNs, mSPNs, and daSNC elements, a decay factor τ, and a coefficient that controls sSPN→daSNC plasticity, κ (eqs. (13),(14),(15),(16)). The weight of a decision-dimension in mSPN, $S_{i,P}(t)$, occurs dynamically depending on whether or not dopamine release is above a specified threshold (eq. (30)). See ***Instance 3: full connectivity and dynamics,*** **Methods**.

c, Modeled action values for the cost-benefit conflict task where an incremented reward (from a low reward of 0 to a large reward of 1 arb. u.) is accompanied by a constant cost (set to 0.25 arb. u.). Increased dopamine leads to increased action value of high-reward, high-cost options and decreased "inaction value," thus leading to more approaches when there is high reward and high cost.

d, The effects of the direct versus indirect pathways are examined in the cost-benefit conflict task used in **Figs. 3 and 4**. Modeled action values change when dmSPN and dsSPN or imSPN and isSPN are inactivated during a task with incremented reward (from a low reward of 0 to a large reward of 1 arb. u.) with medium cost (set to 0.5 arb. u.). Inactivated dmSPNs and dsSPNs lead to lower action values and inactivated imSPNs and isSPNs lead to larger action values.

e,f, Modeled deliberation time for the cost-benefit conflict task is shorter when there is more dopamine. Choice is modeled from action values calculated in the cost-benefit conflict task (here, reward = 1 arb. u., cost = 0.5 arb. u.). The method to derive choice from action values (upwardsloping drifts) and inaction values (downward-sloping drifts) is shown in **e** and modeled deliberation times are shown in **f**.

g-i, The model predicts that dopamine biases actions that contain rewards in physical and/or conceptual proximity. Decision-dimensions important for some decisions but not all will be used to derive action values when there is high dopamine (**g**) but not when there is low dopamine (**h**). If these decision-dimensions correspond to information about location, for instance, then

additional dopamine may lead to the incorporation of spatial information in decisions, leading to actions containing the same location information having more similar action values (**i**).

j-l. Times during learning when the decision-space may undergo a rapid transition, related to **Fig. 5i**. **j.** In the classical model of reinforcement learning encoding by the basal ganglia, dopamine release to the striatum encodes the prediction error, or the difference between expected and observed reward. Instead, in the decision-space model, dopamine communicates the interpretation of the input information (that is, the decision-space), affecting the action taken. **k.** To demonstrate the differences between these models, we designed a simple reinforcement learning task where an agent chooses between two arms of a T-maze task, similar to the costbenefit conflict task used in **Figs. 3 and 4**. The agent learns the reward (+1, chocolate milk) or cost (-1, light) on either end of the T-maze, updating its internal model of the environment based on eligibility traces. Suddenly, at episode 10 (dashed line), the cost and reward switch positions. The agent continues to update its model, changing its pattern of choice after a few sessions. It chooses the highest-value reward/cost option (based on its internal model) 50% of the time, and 50% of the time explores randomly either option. **l.** Reward prediction errors for the right (blue) and left (orange) arms of the T-maze are derived from the Bellman equation (see **Shifts in the decision-Space in a reinforcement learning task, Methods**). Vertical bars show the episodes at which the agent is surprised to find an unexpected dimension and thus would be expected to rapidly transition to a different decision-space. This happens: 1) when the reward is first observed (“to reward”), 2) when the cost is first observed (“to cost”), 3) when the reward is expected but the cost is observed (“reward to cost”), and 4) when the cost is expected but the reward is observed (“cost to reward”). In other words, the decision-space shifts when the animal is surprised to find a decision-dimension they did not expect. These shifts in the decision-space tend to coincide with times when the magnitudes of RPEs are the highest. Thus, the classical RPE theory of dopamine might make similar predictions about dopamine during this task as the decision-space model, even if the underlying functional role of dopamine is different between the models.

(26) $$\tau \cdot \frac{\mathrm{d}s_{\mathrm{sSPN},i,P}(t)}{\mathrm{d}t} = -s_{\mathrm{sSPN},i,P}(t) - x_{i,P}(t) - w_{\mathrm{daSNC}\rightarrow\mathrm{sSPN},i,P} \cdot \left(y_{\mathrm{sSPN},i,P}(t) - \frac{1}{2}\right)$$

(27) $$\tau \cdot \frac{\mathrm{d}s_{\mathrm{mSPN},i,P}(t)}{\mathrm{d}t} = -s_{\mathrm{mSPN},i,P}(t) + x_{i,P}(t) + w_{\mathrm{daSNC}\rightarrow\mathrm{mSPN},i,P} \cdot \left(y_{\mathrm{sSPN},i,P}(t) - \frac{1}{2}\right)$$

(28) $$\frac{\mathrm{d}}{\mathrm{d}t} w_{\mathrm{sSPN} \rightarrow \mathrm{daSNC}, i, P}(t) = \kappa \cdot s_{\mathrm{sSPN}, i, P}(t)$$

where:

(29) $$y_{\mathrm{sSPN}, i, P}(t) = \frac{1}{1 + \exp\left(w_{\mathrm{sSPN} \rightarrow \mathrm{daSNC}, i, P}(t) \cdot s_{\mathrm{sSPN}, i, P}(t) + \mathrm{RMTg} - z_{\mathrm{daSNC}, i, P} \right)}$$

(30) $$\mathrm{S}_{i,P}(t) = \begin{cases} 0 & y_{i,P}(t) < \mathrm{threshold} \\ 1 & y_{i,P}(t) \geq \mathrm{threshold} \end{cases}$$

Supplementary Fig. 8: Individual decision-making differences can be explained by differences in decision-space, Related to Fig. 6.

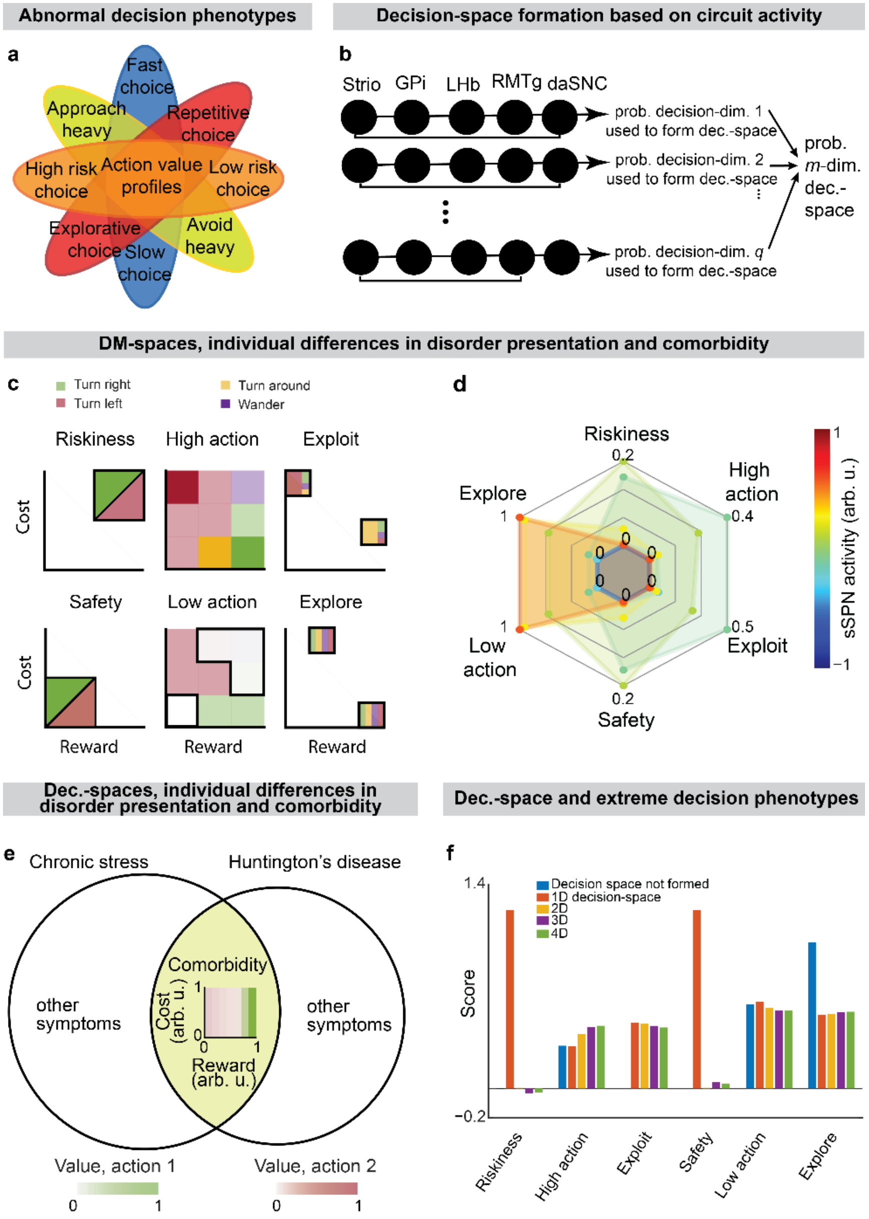

a, Decision-making symptoms observed in disorders.

b, Schematic of the computational model for simulating decision-space based on circuit activity in the analyses plotted in **Fig. 6a**. daSNC activity $\text{daSNC}_1 = \text{daSNC}_2 = \ldots = \text{daSPN}_q = d$ is calculated for each of FSI_C elements, each corresponding to a decision-dimension. Decision-space dimensionality is determined from the Poisson binomial distribution of individual

decisiondimension probabilities, per eq. (54). See **Defining mSPN activity and the decision-space, Methods.**

c, Illustration of the method by which we score subjective valuations along six axes (riskiness, safety, high action, low action, exploit, explore) for a modeled T-maze task. A grid of action values for a range of reward and cost combinations is formed. The “riskiness” and “safety” axes are calculated based on the action values for the high-reward high-cost combinations and low-reward low-cost combinations, respectively. “High action” and “low action” axes are calculated as the proportion of the reward/cost grid with especially high (sum of all action values > 0.5) or low (< 0.2) action value, respectively. The “exploit” and “explore” axes are determined as the proportion of the reward/cost grid with especially high (> 0.5) or low (< 0.25) Gini coefficients between the action values.

d, Extension of **Fig. 6b**. sSPN activity is modified, leading to decision-spaces formed at different rates. These different decision-spaces lead to different action valuations. Thus, in a disorder which affects sSPN activity during decision-making, differences in decision-space may be responsible for differences in decision-making.

e, Huntington’s disease and chronic stress both have decision-making signatures of lowdimensional decision-spaces.

f, Extension of **Fig. 6e**, showing that scores along the six subjective valuation axes (mean taken over 1000 simulations) are different depending on decision-space. Thus, disorders that affect decision-space formation may lead to shifts in decision-making.

(54) $P(m \text{ decision-dimensions used to form decision-space}) = \binom{q}{m} d^m (1-d)^{q-m} \quad m = 0,1,\ldots,q$

Supplementary Fig. 9: Disorder development and decision-space formation, Related to Fig. 7.

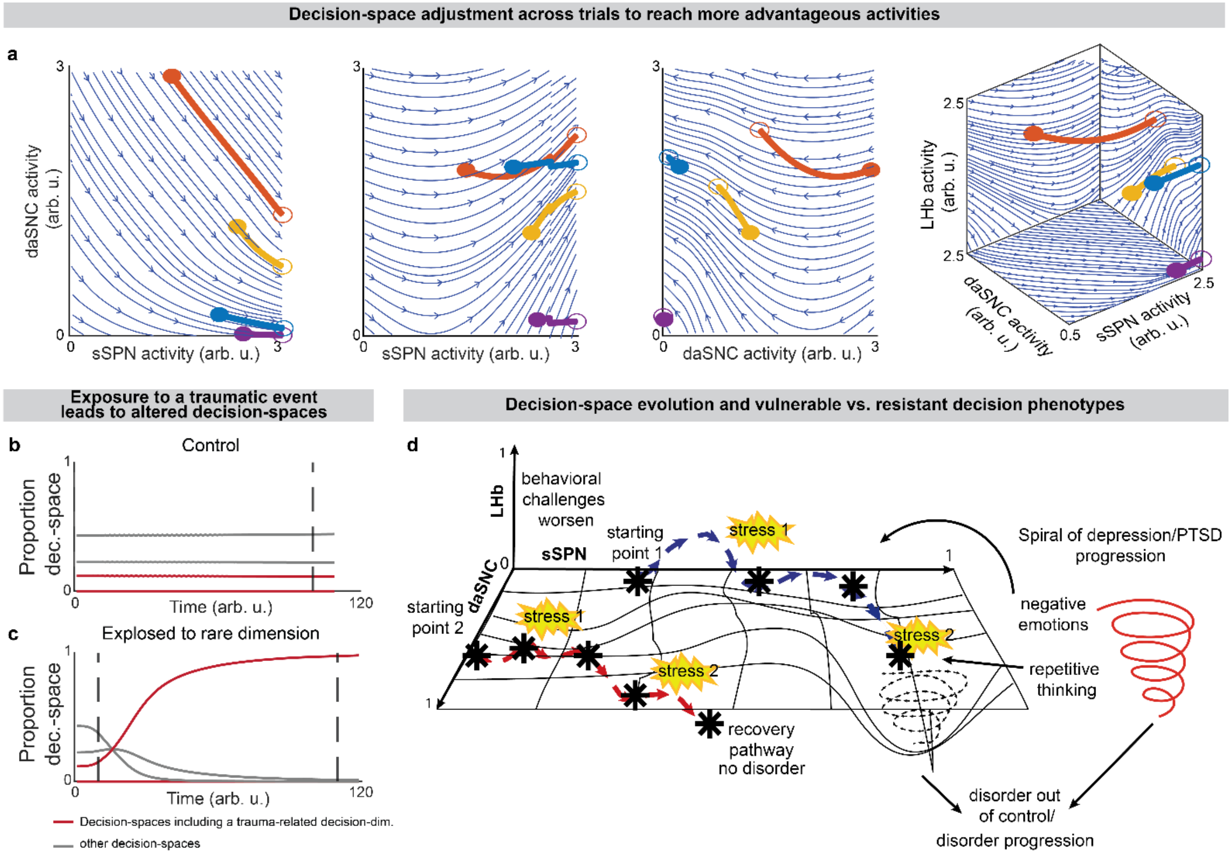

a Simulations of trajectories of circuit activity between trials. Four trajectories of circuit activity movement (thick lines) are plotted above streamlines (blue lines) for a modeled circuit that adjusts to facilitate simple choices. Filled circles are various initial circuit activities (t_0). Empty circles are the ending circuit activities (i.e. t_t). The left three panels are two-dimensional slices of the plot on the right. Here and in **Fig. 7**, the modeled circuit adjusts between trials to improve its ability to form preferred decision-spaces (advantage) while avoiding large changes to activity during decisions (cost). Advantage is represented mathematically as a weighted sum of probabilities that the possible decision-spaces form, given the circuit elements $\{X_1, X_2, \ldots, X_n\}$ take a specified set of activities $x_1, x_2, \ldots, x_n$ (eq. (35)). Cost is represented as the distance between the circuit activity used during decision-making and a "baseline" circuit activity that the circuit takes outside of decision-making (eq. (37)). Net advantage is the difference between advantage and weighted cost (eq. (38)). The circuit adjusts between trials in the direction of maximal change in net advantage (eq. (39)). See ***Movement of circuit activity across multiple trials*, Methods**.

b,c, A possible explanation for the observation that in certain psychiatric disorders (post-traumatic stress disorder and substance use disorder) exposure to a traumatic event or drug can lead to increasingly altered choice after an extended period of nonexposure or abstinence. This can be modeled as adaptation in the circuit to process a rare decision-dimension that was necessary to process, for example, the traumatic event or drug. In the simulation, the circuit's preference for various decision-spaces is updated over time based on their success in making choices according to environmental stimuli. In the disorder resilient scenario (**b**), the circuit is first exposed to the traumatic event or drug at the dashed line. The "red" decision-space is not formed as often as are other decision-spaces (gray lines). In contrast, even short periods of exposure to the traumatic event or drug can impact future decision-spaces, resulting in vulnerability (**c**). Circuit activity adjusts until, when the traumatic event or drug reappears, the "red" decision-space forms frequently. This result may explain incubation of fear (in post-traumatic stress disorder) or craving (in a substance use disorder), where symptoms emerge only after a period of weeks after exposure. Differences in response post-incubation lead to modeled vulnerability or resilience.

d, Circuit activity morphs over time in response to decision-making needs.

(35) $$\text{advantage}(X_1=x_1, X_2=x_2, \ldots, X_n=x_n) = \sum_{l=1}^{2^q} \text{score}_l \cdot \text{P}(\text{space}_l | (X_1=x_1, X_2=x_2, \ldots, X_n=x_n))$$

(37) $$\text{cost}(X_1=x_1, X_2=x_2, \ldots, X_n=x_n) = \left\| \begin{bmatrix} x_1 & x_2 & \cdots & x_n \end{bmatrix}^\intercal - \begin{bmatrix} x_{1,\text{baseline}} & x_{2,\text{baseline}} & \cdots & x_{n,\text{baseline}} \end{bmatrix}^\intercal \right\|_2$$

(38) $$\text{net advantage}(X_1=x_1, \ldots, X_n=x_n) = \text{advantage}(X_1=x_1, \ldots, X_n=x_n) - \text{constant} \cdot \text{cost}(X_1=x_1, \ldots, X_n=x_n)$$

(39) $$\frac{\Delta \begin{bmatrix} x_{1,\text{baseline}} & x_{2,\text{baseline}} & \cdots & x_{n,\text{baseline}} \end{bmatrix}^\intercal}{\text{trial}} = \text{rate} \cdot \nabla \text{ net advantage}(X_1=x_1, \ldots, X_n=x_n)$$

Supplementary Fig. 10: Proposed experiments that might add support to our model.

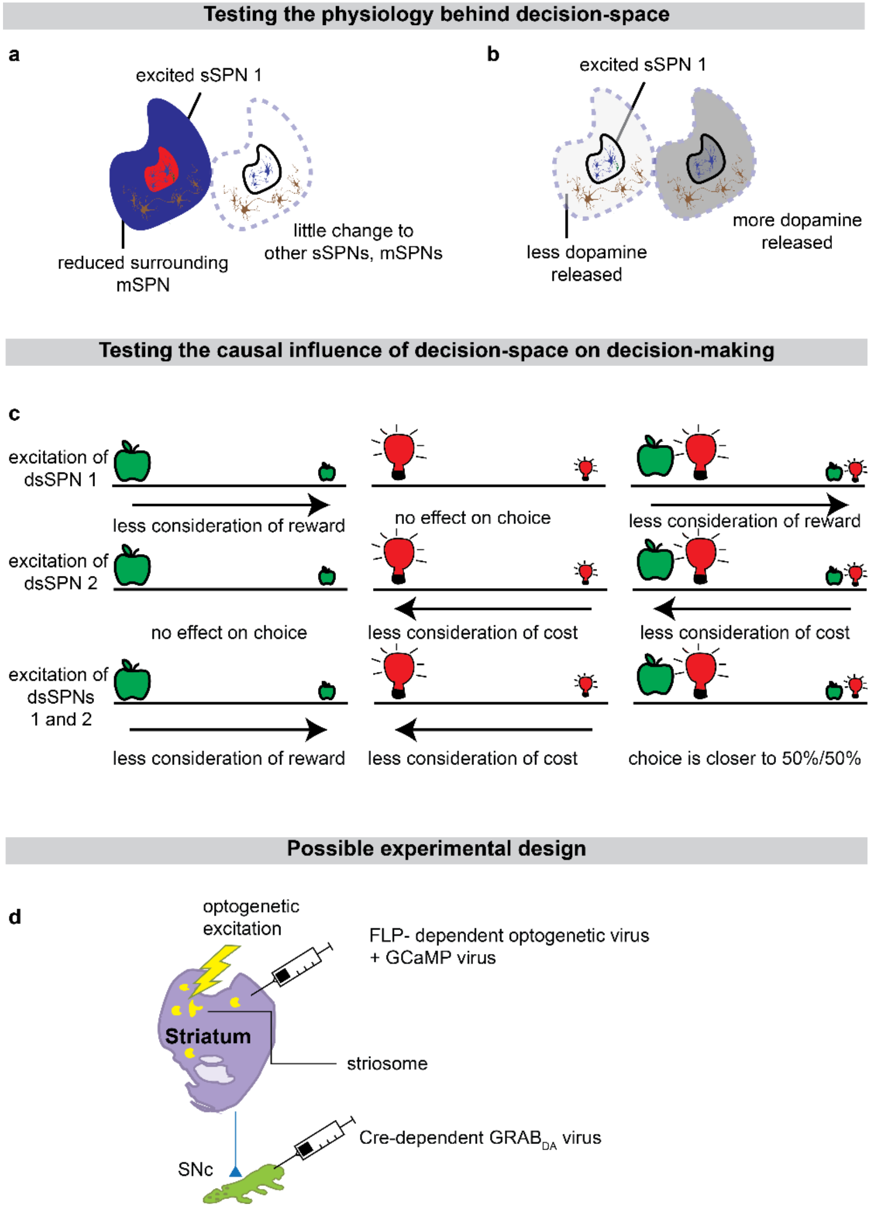

a, The decision-space model assumes that when an sSPN subpopulation is active, the corresponding decision-dimension is unlikely to be used to form the decision-space. Thus, activating an sSPN subpopulation (red) should lead to lower activity in neighboring mSPNs (blue) and there should be lower variance in their activities. This would support our hypotheses that sSPNs bias a nearby mSPN subpopulation towards being used or excluded during the formation of decision-space.

b, Meanwhile, we would expect to find less dopamine (light gray versus darker gray) released to the sSPN subpopulation and neighboring mSPNs than to other SPNs. This would support our hypothesis that the decision-space is formed via selective dopamine release.

c, We would expect sSPN stimulation to alter decision-making. An experiment could be designed asking rodents to choose rewards (apple) and/or costs (lights) in a T-maze (line, animal begins in the center). The rodents would be expected to selectively deprioritize informational dimensions corresponding to sSPN subpopulations that are stimulated. For instance, stimulating rewardresponding sSPNs would be expected to lead to reduced consideration of reward when making the decision.

d, The hypotheses in **a-c** can be tested with tools available to neuroscientists. Similar to was performed by Lazaridis et al. (2024), DA-Cre-expressing mouse are crossed with striosomal-FLPexpressing mice. Three viruses are injected: 1) Cre-dependent $GRAB_{DA}$ into daSNC (for instance, similar to Sun et al. (2020))[67], 2) FLP-dependent optogenetic virus into the striatum, 3) general GCAMP virus into the striatum. Striosome and matrix are then recorded using two-photon microscopy, similar to, for example, Bloem et al. (2022). Four colors are used to: 1) identify dopamine via the $GRAB_{DA}$ virus 2) identify all striosomal and matrix neurons via the GCaMP virus, 3) identify the contours of the striosomes via the FLP-dependent virus, and 4) stimulate the striosome. In a closed loop way, striosome neurons are identified that correspond to reward or cost, and the neurons that selectively respond to each are stimulated as rodents perform a Tmaze task that was used by, for instance, Friedman et al. (2015).

Supplementary Tables.

Supplementary Table 1: Evidence for the connectivity used in our model.

Connectivity	Evidence
cortex→sSPN/mSPN	• Research has found that nearly, if not all cortical regions project to the striatum[1,2], however only a subset of cortical areas have been determined to be compartment specific[3,4], with examples of those listed below.<br>• Evidence that both the striosomes and matrix receive input from sensorimotor, limbic, and associative regions[5].<br>• Evidence that several regions (prelimbic cortex, infralimbic cortex, posterior orbitofrontal cortex, insular cortex) project more to striosomes than matrix[6,7].<br>• Evidence that cortical regions (primary motor cortex) project more to matrix than to striosomes[8].
cortex→FSI→sSPN/mSPN	• Evidence for FSI connections to both striosomes and matrix[9–11].
sSPN (more so than mSPN)<br>→daSNC	• Range of evidence from primates, rats[12,13], and mice[8] suggesting a stronger connection striosome connection than matrix.<br>• One study suggests that matrix also projects to daSNC[14].
sSPN (more so than mSPN)<br>→GPi→LHb→RMTg→daSNc	• Range of evidence supporting the striosome to EP (nonprimate GPi correlate) connection in rats[12,15].<br>• Evidence supporting the striosome to GPi connection in primates[16].<br>• Evidence of the EP to LHb connection in rats[17].

	• Evidence that striosomes more so than matrix drive striatal influence on LHb activity[18], potentially through pallidal regions[19].<br><br>• Evidence in primates[17], rats[20] , and mice[19] of the LHb to RMTg (also called tVTA) connection.<br><br>• Evidence that the RMTg projects to daSNc[21,22].
sSPN (more so than mSPN)<br>→GPe→daSNC	• Evidence of the striosome to GP (non-primate GPe correlate) but not matrix to GP connection in mice[23] and rats[12].<br><br>• Evidence of a striosome (more so than matrix) to GPe to daSNC pathway[23].
daSNC→sSPN/mSPN	• Evidence that dopamine is released to both compartments, with faster[24] and more[25,26] dopamine release to striosomes than matrix.

Supplementary Table 2: Criteria used in Supplementary Tables 3-7 to test our decision-space model and compare it to alternative models of the circuit.

Modeled Functional role of a circuit region.	Expectation per the model.
**sSPNs influence the priority of decision-dimensions, thereby affecting decision-space.** **(decision-space model)**	**1.A.** During a high-dimensional decision-space, choice more closely aligns to experimental inputs (e.g. chocolate milk level, light brightness) during difficult tasks (e.g. consideration of both rewards and costs).
	**1.B.** A low-dimensional decision-space is often formed from decisiondimensions which are commonly used during a decision-making task, for instance information about reward in rodents that are trained to respond to reward cues.
	**1.C.** Cortical data is mapped orthogonally and continuously onto SPNs.
**sSPNs encode conflict.** **(our conflict model)**	**2.A.** sSPN activity scales with conflict between two features like reward and cost.
	**2.B.** Changes to conflict, revealed by sSPN activity, alter choice.
	**2.C.** Changes to sSPN signals are greatest when conflict is introduced.
**sSPNs encode subjective values.** **(our subjective value model)**	**3.A.** sSPN activity scales (possibly directly or inversely) with subjective value of stimuli, likely roughly tracking reward minus cost.
	**3.B.** Higher subjective value, reflected by sSPN activity, leads to increased selection of an offer.
	**3.C.** Changes to sSPN activity are strongest at the time during scenarios where cues are associated with subjective values.
**sSPNs encode prediction errors.**	**4.A.** sSPN activity should change proportionally and continuously to the difference between expected and actual reward and cost at each time step.

**(our prediction error model)**	**4.B.** As a task is learned, the trend in sSPN activity over time changes as earlier cues become associated with later outcomes.

**sSPNs encode actions.**	**5.A.** Different neurons would encode different actions
	**5.B.** Activity of sSPN subpopulations should scale directly or inversely with the predisposition of action execution.
**(our actions model)**	**5.C.** Changes to the signals of action-encoding subpopulation would be greatest prior to or during action execution.
**GPi during decision-space formation.**	**6.A.** Changes to activity along a decision-dimension is reflected in GPi activity.
	**6.B.** A given GPi neuron encodes data across multiple decisiondimensions.
**(decision-space model)**	**6.C.** Activation of GPi causes more decision-dimensions to be incorporated into the decision-space while inactivation causes fewer decision-dimensions to be used.
**LHb and RMTg optimizing or modifying decision-space.**	**7.A.** Active LHb (or RMTg) leads to choice reflective of reduction in dimensionality of the decision-space and vice versa.
**(decision-space model)**	**7.B.** LHb (or RMTg) is active during times when lower-dimensional decision-spaces are beneficial to decision-making.
**Dopaminergic neurons of the SNc during decision-space formation.**	**8.A.** There exist subpopulations of daSNC neurons that encode information along an orthogonal axis of information.
**(decision-space model)**	**8.B.** Activity in one daSNC subpopulation only affects the subpopulation of SPNs corresponding to one decision-dimension.
**Direct and indirect pathways alter decision-space formation.**	**9.A.** Higher dopamine leads to lower dimensionality of the direct pathway decision-space while lower dopamine leads to higher dimensionality, and vice versa for the indirect pathway.

**(decision-space model)**	**9.B.** Direct pathway mSPNs promote actions while indirect pathway mSPNs aid in action suppression.
	**9.C.** Subpopulations of mSPNs encode data along decisiondimensions orthogonally and continuously over time.

Supplementary Table 3: Testing alignment of the decision-space model and other models to a selection of the experimental sSPN literature.

	Criterion	**Friedman et al. (2015)[9] (I)**	**Friedman et al. (2020)[10] (II)**	**Bloem et al. (2022)[27] (III)**	**Xiao et al. (2020)[28] (IV)**	**glage et al. (2021)[29] (V)**
Decisionspace model	1.A	✓	✓	∅	≈	∅
	1.B	∅	✓	∅	∅	∅
	1.C	∅	∅	✓	✓	✓
Conflict model	2.A	✓	×	×	×	∅
	2.B	✓	∅	∅	∅	∅
	2.C	✓	×	×	×	∅
Subjective value model	3.A	×	✓	×	×	×
	3.B	✓	✓	∅	✓	×
	3.C	✓	✓	✓	✓	×
Prediction error model	4.A	×	×	✓	×	×
	4.B	∅	×	✓	✓	∅
Actions model	5.A	∅	∅	✓	✓	≈
	5.B	✓	✓	✓	✓	≈
	5.C	≈	✓	✓	✓	≈

✓ -- aligned with criterion

≈ -- somewhat aligned to criterion

× -- not aligned with criterion

∅ -- experiment does not test criterion

1.A.I. Striosome activation led to less consistent choice in tasks that required processing of both reward and cost, while sSPN inhibition led to more consistent choice in tasks that required processing of both reward and cost. Decision-making was less affected by sSPN manipulation in tasks that required processing either reward or cost, but not both. Meanwhile, in the absence of manipulation, the rodents had less active sSPNs during tasks that required processing both reward and cost. These results may suggest that a higher-dimensional decision-space (formed at low striosome activity) is associated with processing of multiple informational dimensions in a consistent way.

1.A.II. Rodents that best learned the reward/cost cue discrimination task had high sSPN activity after the reward cue and low sSPN activity after the cost cue. The rodents that learned less well had similar activity between the tasks. This may suggest that the most consistent choices, made by the rodents that learned, were formed using a context-dependent decision-space.

1.A.IV. Inhibition of Tfz1 neurons, which were shown to encode either reward or cost independently, decreased accuracy and correct rejection rate in cost trials. This may suggest a change to the priority assigned to the reward and cost decision-dimensions. Notably, the direction of the effect is opposite what the model expects, if only cost-related SPNs were inhibited. However, Tfz1 neurons that responded to rewards and costs were inhibited simultaneously, making for a less clear model prediction on the direction of the effect.

1.B.II. More rodents successfully learned the reward task than the cost task, and this was reflected in fewer rodents forming a reduced sSPN activity and reaching a putative high-dimensional decision-space. This may suggest that most rodents formed a one-dimensional reward-related decision-space and only the rodents that learned formed, in the appropriate context, a twodimensional reward-and-cost-related decision-space.

1.C.III. In the probabilistic bandit task, dynamic changes along the orthogonal information axes of reward and cost led to proportional changes in sSPNs that resembled prediction errors. Some of the identified neurons responded only to reward or only to cost. This may suggest that those neurons are members of subpopulations corresponding to a reward-predominant decisiondimension or a cost-predominant DM-dimension.

1.C.IV. Tfz1-expressing sSPNs showed evidence of encoding reward or cost dimensions independently (although it was not confirmed that Tfz1 neurons were responding to cortical signals). During a real-time place preference task, different neurons were activated by either

reward or cost individually both during administration of the reward or cost and at a cue associated with the reward or cost. This may suggest encoding of information along decision-dimensions, including related information observed from separate stimuli.

1.C.V. During a multiphase task that required attention to many stimuli and strategies, sSPN and mSPN activities in general followed somewhat similar activity patterns over time. The most significant indicator of SPN activity was task phase. This may suggest a continuous mapping, with similar weights between sSPN and mSPN, of cortical information relevant to the current phase of the task onto the decision-space in the striatum.

2.A.I. Mean sSPN activity was significantly different in the task where there was the most conflict than in the other tasks.

2.A.II-IV. Conflict was not introduced experimentally, yet sSPN activity was different between tasks.

2.B.I. Optogenetic manipulation of striosomes, which may alter the level of encoded cost/reward conflict, altered choice.

2.C.I. In the task, conflict is important to the rodent when it determines which option to select, which is when striosome activity rises. This may suggest that striosomes encode conflict.

2.C.II-IV. Conflict was not introduced experimentally, yet sSPN activity spiked when non-conflict stimuli were introduced.

3.A.I. sSPN activity did not scale, directly (more sSPN activity, more subjective value) nor inversely (more sSPN activity, less subjective value), with either the overall values of the options or with the difference between the value of the options on the right arm of the T-maze versus the left arm. sSPNs had lowest mean activity in the cost-benefit conflict task, which had a moderate difference between reward and cost. Both the benefit-benefit task, which had high reward and no cost, and the cost-cost task, which had low reward and high cost, were performed with high sSPN activities.

3.A.II. sSPN activity was higher during the reward task than the cost task among rodents that learned the task. This may suggest that sSPN activity tracks subjective value of the presented

stimuli, with the reward-related stimuli being assigned higher subjective value than the costrelated stimuli.

3.A.III. The activities of a sizable subpopulation of SPNs were found to track reward level and cost level separately, not together as our subjective value model would expect. Meanwhile, neurons that tracked both reward and cost had activities that did not scale with reward minus cost, suggesting encoding of information other than the subjective value of the stimuli.

3.A.IV. Separate SPN subpopulations were found to track reward or cost level, suggesting encoding of reward and cost separately, rather than together as our subjective value model would expect.

3.A.V. There was not a strong relationship between rewards minus costs presented in the different task phases and sSPN activity.

3.B.I. The rodents approached the higher-reward option most in the task (cost-benefit conflict task) which had the lowest sSPN activity, suggesting a possible relationship between subjective value assigned to the left and right arms of the T-maze and sSPN activity.

3.B.II. The choice to lick was made more frequently when sSPN activity during the licking period was higher.

3.B.IV. Stimulation and inhibition of Tfz1-expressing neurons led to opposite effects on decisionmaking, suggesting that manipulation may change subjective values assigned to stimuli.

3.B.V. There was not a strong relationship between sSPN activity and choice in the multiphase task.

3.C.I. Across all tasks, sSPN activity ramped during the period of the decision where a choice was made as to which offer to approach, which is also likely the period of the task that most requires an assignment of subjective value to environmental stimuli. This suggests that perhaps sSPNs play a role in assigning subjective value.

3.C.II. sSPN activity rose during the periods when the rodents chose whether to lick, which is the period of the task that likely most requires an assignment of subjective value to environmental stimuli. This suggests that perhaps sSPNs play a role in assigning subjective value.

3.C.III. sSPN activity was the highest at the cue and during the outcome period, two intervals when it is likely important to assign subjective value to stimuli. This suggests that perhaps sSPNs play a role in assigning subjective value.

3.C.IV. sSPN activity rose during the cue and administration periods of the Pavlovian conditioning task, both of which are likely important for assignment of subjective value. This suggests that perhaps sSPNs play a role in assigning subjective value.

3.C.V. sSPNs did not have exceptionally high nor low activities during periods of the multiphase task in which we might expect subjective value assignment to be important. sSPN activity was higher during a locomotion phase, for example, than during a phase where a reward was presented.

4.A.I. The rodents in the experiments were overtrained, task types were randomized, and each task type had different levels of reward and/or cost. Therefore, they might be expected to experience prediction error when they entered the maze and become aware of the task type. Prediction upon entering the maze should be roughly equivalent to the average value (reward minus cost) of all tasks. By this logic, there is positive prediction error in tasks where the expected outcome was greater than the expected outcome upon entering the maze, and vice versa. Thus, the largest positive prediction error likely occurred during the benefit-benefit task, followed by the cost-benefit non-conflict task, followed by the cost-benefit conflict task, followed by the cost-cost task. Experimental sSPN activities were not ordered like this.

4.A.II. Theoretically, we would expect, post-learning, a prediction error at the tone because the value of the session changes at this point. Yet sSPN activity had little change at the tone postlearning.

4.A.III. The authors demonstrated that separate populations track prediction error directly and inversely.

4.A.IV. Theoretically, prediction error moves from the administration period to the cue during a Pavlovian task. This is not what was observed: sSPN activity spiked during the administration period just as much after learning as before.

4.A.V. sSPN activity had little change at periods of the task when prediction errors were introduced experimentally.

4.B.II. sSPN activity over time did change with learning but spikes in activity did not develop at times (e.g. the cue) we would expect post-learning prediction errors to develop.

4.B.III. sSPN activity tracked prediction error more closely at the cue after learning than before. This suggests that sSPNs may be encoding prediction error and learning the association between the cue and the outcome.

4.B.IV. The activities of individual SPN increased in magnitude at the cue after learning in the Pavlovian conditioning task (although there was not, as our prediction error model would expect, a corresponding reduction during administration). In the active avoidance task, failure-responding sSPNs increased activity upon punishment delivery.

5.A.III. Different neurons were found to respond to reward versus cost. This may suggest that different neurons were involved in encoding actions planned in response to the different stimuli.

5.A.IV. Different neurons were active in response to reward versus cost. This may suggest that different neurons were involved in encoding of actions planned in response to the different stimuli.

5.A.V. Neurons encoded actions in task-specific contexts but remapped between tasks.

5.B.I. The optogenetic manipulation caused altered actions (turn right versus turn left), suggesting that perhaps stimulation or inhibition leads to upweighting of one action versus another.

5.B.II. Different actions (licking versus non-licking) corresponded to different mean sSPN activity. This may suggest that sSPNs encode the action of licking.

5.B.III. sSPN subpopulations changed in response to unexpected stimuli, perhaps to recalculate actions to take.

5.B.IV. The reward-active sSPNs were most active during the outcome period and less so during the cue. This could be a sign of a potential action linked to the stimuli.

5.B.V. sSPN subpopulations were identified but their activities were only related to actions on a task-by-task basis.

5.C.I. For many trials, sSPN activity increased during the period when the animal made a choice between turning left versus turning right. However, sSPN activity was roughly constant throughout the choice periods of cost-benefit conflict trials, and the actions model would expect a ramping of activity here, too.

5.C.II. sSPN activity increased during the period when the animal made a choice whether or not to lick. This may suggest that sSPNs encode the action of licking.

5.C.III. sSPN subpopulations responded to the prediction error. Through the lens of the action model, perhaps sSPNs are revising the potential actions that will be initiated.

5.C.IV. sSPN activity was positively correlated with running velocity, and sSPN activity ramped along with licking bouts throughout the cue and outcome periods. This suggests that perhaps sSPNs encode the action of running.

5.C.V. sSPN subpopulations were most active when certain actions (e.g. turn direction) occurred, but between tasks these were not responsive during the same actions.

Supplementary Table 4: Testing the alignment of the decision-space model to a selection of the experimental literature on GPi.

Criterion	**Weglage et al. (2022)[19] (I)**	**Munte et al. (2017)[30] (II)**	**Stephenson-Jones et. al (2016)[31] (III)**
6.A	∅	✓	∅
6.B	∅	∅	✓
6.C	✓	∅	∅

✓ -- aligned with criterion

≈ -- somewhat aligned to criterion

× -- not aligned with criterion

∅ -- experiment does not test criterion

6.A.II. Level of reward correlated with GPi activity. This may suggest that GPi activity scales up or down depending on the level of information along a reward-related decision-dimension.

6.B.III. Individual LHb-projecting GPi neurons were both excited by punishment-predicting cues and the punishment itself and were inhibited by rewards and their associated cues. This may suggest that information along two decision-dimensions, one reward-related and one-cost related, is encoded by the same GPi neurons. Further, the opposite response of GPi neurons to reward and cost lends support for our choice to differentially weight GPi inputs from sSPN subpopulations corresponding to different decision-dimensions.

6.C.I. An identified subpopulation of LHb-projecting GPi affected the profile of choices made, aligning with the functional role of the GPi in our model. Decreased activity of these neurons was associated with increased commitment to actions, which may correspond to effective formation

of a decision-space. This would align with the hypothesis of the model that lower GPi activity leads to a higher-dimensional decision-space.

Supplementary Table 5: Testing the alignment of the decision-space model to a selection of the experimental literature on LHb and RMTg.

Criterion	Matsumoto & Hikosaka (2007)[32] (I)	Lee & Hikosaka (2022)[33] (II)	Stopper & Floresco (2014)[34] (III)	Vento et al. (2017)[35] (IV)
7.A	∅	∅	≈	✓
7.B	✓	✓	∅	∅

✓ -- aligned with criterion

≈ -- somewhat aligned to criterion

× -- not aligned with criterion

∅ -- experiment does not test criterion

7.A.III. LHb inactivation led subjects to change their choice during a probabilistic discounting task to accept a large, risky reward over a smaller, safe reward. As our model expects, LHb inactivation played an important role in affecting decisions that required multiple decision-dimensions. It is expected, however, that reduced LHb activity produces enhanced adherence to any decisiondimensions required to perform the task. The subjects with inactive LHb would appear to be incorporating fewer, not more, decision-dimensions into their choices. One possible explanation is that LHb inactivation led to an overwhelming increase in dimensionality of the decision-space that reduced focus on a few important decision-dimensions, such as reward.

7.A.IV. RMTg selectively altered decisions, primarily in response to cost. This may suggest that RMTg inactivation led to choices with less adherence to an important cost-related decisiondimension.

7.B.I. LHb activation led to suppression of dopaminergic signaling among daSNC neurons. LHb was active at times when it may not have been beneficial to construct a decision-space involving a reward-related decision-dimension (when no reward was presented) but inactive when it may have been beneficial to construct a decision-space using a reward-related decision-dimension (when reward was presented).

7.B.II. LHb was found to alter its activity depending on situational context. LHb was most active at times when it may not have been beneficial to construct a decision-space involving a rewardpredominant decision-dimension (when it was indicated that minimal reward would be available or when less than expected reward was presented) and at times when evaluation of data along decision-dimensions may have been less necessary (the uncontrollable tasks).

Supplementary Table 6: Testing the alignment of the decision-space model to a selection of the experimental literature on daSNC.

Criterion	**Fiorillo et al. (2003)[36] (I)**	**Matsumoto & Hikosaka (2009)[37] (II)**	**Gan et al. (2010)[38] (III)**	**Bromberg-Martin et al. (2010)[39] (IV)**	**Kim et al. (2020)[40] (V)**	**Long et al. (2024)[41] (VI)**
8.A	✓	✓	✓	✓	✓	∅
8.B	∅	∅	∅	∅	∅	≈

✓ -- aligned with criterion

≈ -- somewhat aligned to criterion

× -- not aligned with criterion

∅ -- experiment does not test criterion

8.A.I. daSNC neurons responded differently during the cue and during the outcome period depending on the likelihood of a cue predicting a reward outcome. This may suggest that a subpopulation of daSNC neurons encodes information along a reward-related decisiondimension, and probabilistic inputs are reflected continuously over time as the information along the decision-dimension is updated.

8.A.II. Two daSNC populations responded very differently to rewarding or aversive stimuli and a third group was non-responsive. This may support the tenet of the decision-space model that different daSNC subpopulations correspond to different decision-dimensions, some of which might be related to reward information, some to cost information, and some to neither reward nor cost information.

8.A.III. The activities of recorded dopamine neurons showed more resemblance to reward levels than to overall utility. This may support the tenet of the decision-space model that different dopamine subpopulations correspond to different decision-dimensions, some of which are related to reward information, and that dopamine neurons encode data along decision-dimensions, not an overall value function.

8.A.IV. Subpopulations of daSNC neurons that encoded value were excited by rewarding information while salience neurons were excited by both rewarding and aversive cues. This may support the tenet of the decision-space model that different daSNC subpopulations correspond to different decision-dimensions, some of which are related to reward information and some to other information.

8.A.V. Dopamine changed in response to altered proximity to reward. This may support the architecture of the decision-space model, where changes to reward information are captured in an sSPN subpopulation related to reward, then passed to a corresponding daSNC subpopulation.

8.B.VI. VTA cells were optogenetically inhibited or excited as ventral striatal neurons were recorded. Several findings are particularly relevant to the decision-space model: 1) A subpopulation of the striatal neurons responded to reward, and the activities of these neurons correlated with the VTA neurons. 2) 8% of all SPNs (4% above control, both non-rewardresponding and reward-responding) had altered activities when VTA was inhibited. 3) The physical location of the SPNs that had altered activities had significantly distinct locations. Finding 1 may support the decision-space model, where mSPNs and sSPNs receive somewhat similar cortical inputs and sSPNs influence daSNC activity. A proportion of the reward-responding SPNs may encode a reward-related decision-dimension (the others may encode other information about the task and the reward administration). Further, per Finding 2, only a proportion of SPNs were affected by the reward-induced daSNC activity, supporting the selective release of dopamine to a reward-related SPN subpopulation in the model. Per Finding 3, the SPNs that did change their activities had spatial organization, supporting the assumption of our model that SPN subpopulations corresponding to decision-dimensions are organized spatially. The experiments, however, were primarily conducted on VTA neurons, not daSNC neurons, and a decoder did not accurately discriminate inhibition from control trials based on SPN spiking. This might be because a reward-related decision-space would require the modulation of more than 8% of neurons. Per our model, a decision-space would be formed in cases where sufficient dopamine was released

to modulate a larger percentage (but not all SPNs). Indeed, when VTA neurons were manipulated to release more dopamine than they ordinarily did during the head-fixed licking task, up to 37% SPNs responded and a decoder successfully linked firing rates to the task, suggesting the formation of a decision-space. It may be that a decision-space only forms in certain tasks, for instance perhaps in tasks that require action selection, a hypothesis that might be supported by the findings of Samejima et al.[42] and Seo et al.[43] Our model provides a framework for taskdependent dopamine release to be studied in future work.

Supplementary Table 7: Interpreting a selection of the experimental literature on the direct versus indirect pathways through the decision-space model.

Criterion	**Parker et al. (2018)[44] (I)**	**Peak et al. (2020)[45] (II)**	**Maltese et al. (2021)[46] (III)**	**Barbera et al. (2016)[47] (IV)**
9.A	✓	∅	✓	✓
9.B	∅	✓	∅	✓
9.C	∅	∅	∅	✓

✓ -- aligned with criterion

≈ -- somewhat aligned to criterion

× -- not aligned with criterion

∅ -- experiment does not test criterion

9.A.I. The direct and indirect pathway were found to be typically coactivated, and dopamine depletion differentially altered direct versus indirect pathway dynamics. This may support the tenet of our model that a decision-space is formed in each pathway during a decision and the two decision-spaces are affected differently by dopamine.

9.A.III. Increased dopamine release led to increased activation of dSPNs and decreased activation of iSPNs. Decreased dopamine release led to the opposite change. This supports the decision-space model, in which dopamine excites dmSPNs and inhibits imSPNs.

9.A.IV. Cocaine led to the increase in activity of a direct pathway subpopulation and simultaneously a decrease in activity of a neighboring indirect pathway subpopulation. This may

suggest the simultaneous activation of a direct pathway decision-dimension and inactivation of a direct pathway decision-dimension.

9.B.II. Inhibition of dSPNs during learning led to blunted action associations, while inhibition of iSPNs led to a reduced ability to switch actions based on context. This supports the decisionspace model, in which the direct pathway is involved with performing actions and the indirect pathway is involved in refraining from actions.

9.B.IV. Cocaine administration led to increased direct pathway activity, decreased indirect pathway activity, and more movement. This may suggest that the direct pathway more than the indirect pathway is associated with initiating actions.

9.C.IV. Subpopulations encoding data along decision-dimensions showed high intra-cluster synchrony that was stable across days, and inter-cluster synchrony was significantly lower. This may support the tenet of the decision-space model that similar information is encoded within proximate SPN subpopulations.

Supplementary Notes.

Supplementary Note 1: Functional and connectivity differences between striosomes versus matrix.

The inclusion of striosomes versus matrix SPNs in models of the basal ganglia is crucial due to their important differences. First, striosomes and matrix have important functional differences. sSPNs are implicated in multiple decision-making functions including conflict[9], value-based learning[10], changing strategies to suit changes in tasks[29], responding to negative stimuli[28], responding to probabilistic cues/reward prediction errors[27,48], habitual behaviors[49], cognition[50], and motor control[51,52]. In contrast, matrix neurons are important for motor functions[53] along with action selection and initiation[54].

Second, striosomes and matrix have different connectivity. While the striosomes receives convergent signaling from numerous cortical regions[1,2], the prelimbic cortex, infralimbic cortex, posterior orbitofrontal cortex, insular cortex preferentially project to sSPNs[6,7] while matrix receives preferential projections from the primary motor and sensory cortex[8]. Both sSPNs and mSPNs are roughly evenly distributed between the direct pathway, identified by D1 receptor expression, and indirect pathway, identified by D2 receptor expression[4,12,55–58]. Only direct pathway sSPN neurons project to the dopaminergic neurons of the SNc[12] and to GPi[16]→LHb[17]→RMTg[17]→ dopaminergic neurons of the SNc[21,22]. Only indirect pathway sSPNs project to the GPe core which target daSNc neurons[55]. These regions influence behavioral states[3,59], action selection[60–63], and determine subjective valuations[64–66]. The prior mentioned regions associated with the sSPNs direct pathway (LHb, RMTg, dopaminergic neurons of the SNc) respond to aversive/rewarding cues[67–71], are sensitive to reward/cost[72–74], and signal Reward Prediction Errors (RPE)[75–78]. The modeled regions and neuronal subdivisions are differentially affected in neuropsychiatric disorders[6,79–91].

Third, striosomes and matrix differ in which genes they express. Striosomes express *BACH2*, *KCNT1*, *KCNIP1*, and *KHDRBS3* while matrix neurons express *STXBP6*, *GDA*, and *SEMA3E*[92]. Both compartments demonstrate distinct molecular expression patterns[6]. Further, the striosomes and matrix develop over different periods, with striosomes developing earlier than matrix during a period of prenatal development[3].

Finally, the striosomes and matrix are oppositely modulated by direct pathway dopamine receptor activation[93]. In mSPNs, direct pathway activation generates and lengthens an "up-state", a depolarized state where neurons can more readily reach a threshold to fire an action potential. In contrast, sSPNs are more excitable during basal conditions and direct pathway receptor activation shortens up-states and reduces the time in which sSPNs can fire action potential[93]. We model this in **Fig. 5.** Given all these factors, the development of computational models that take account for striosomal and matrix striatal subdivisions are critical to understanding decision-making and learning.

Supplementary Note 2: Choice of circuit elements.

Using a reductionist approach, we selected circuit elements *crucial to the regulation of dopamine by dorsomedial striosomes*. Thus, we prioritized the direct connection between striosomes and daSNC and the regulation of daSNC via GPe and GPi→LHb→RMTg.

Notably, we exclude from our model the substantia nigra reticula (SNr), another basal ganglia region that receives projections from the striosomes[12,94] and the subthalamic nucleus[95] and feeds back to daSNC neurons[96], forming an additional secondary striosome→daSNC connection. In the context of our model, sSPN→SNr→daSNC helps to determine *how many* decision-dimensions are included in the parallel direct and indirect pathway decision-spaces, in conjunction with the modeled operation assigned to the GPi→LHb→RMTg pathway. The connection through SNr, by introducing a striosome→SNr→daSNC→striosome loop, would add an additional set of dynamics on a longer time scale than the modeled striosome→daSNC→striosome loop. The analysis using ***Instance 1*** of the model (full connectivity, no dynamics), however, would remain similar, with similar selective effects of striosomes, GPi, LHb, RMTg, and daSNC on the decision-space and choice.

Other regions that might be included in a future expanded model are the central nucleus of the amygdala, the paraventricular thalamic nucleus, the rhomboid, and the paratenial thalamic nucleus, each of which provides input to the striosomes more so than matrix[14,97–100]. In the context of our model, these connections would help to influence *which* decision-dimensions are included in decision-space, in conjunction with cortical neurons, FSIs, sSPNs, and daSNC neurons.

More broadly, our model does not include certain brain regions that are relevant to decisionmaking such as the dorsolateral striatum, ventral striatum, dopaminergic neurons in the ventral tegmental area (VTA), subthalamic nucleus, and other basal ganglia regions. There are also brain regions outside the basal ganglia that are implicated in decision-making[101] that we do not consider.

We did not consider neuronal molecular heterogeneity or gene expression.

Supplementary Note 3: Choice of cortical inputs to the striatum.

Experimental work has revealed dimensionality reduction on the order of ~100 times from cortex to SPNs[102]. We focus on the processing of information by the striatum rather than the specific information encoded by each region of the cortex. This allows us to retain focus on the processing of information by the striatum. Additionally, our model assumes that cortical inputs are roughly similar between the striosomes and matrix. This assumption allows us to assign the same decision-dimensions to striosome subpopulations as to matrix subpopulations. As shown in **Supplementary Table 1**, in reality, several striatum-projecting cortical regions have been found to project more strongly to either striosomes or matrix[6,7,13], although the experimental data on many of these regions is mixed[14]. However, we suggest that there are somewhat similar representations of decision-dimensions in sSPNs and mSPNs. Computationally, as shown in **Supplementary Fig. 5**, average population activities can successfully encode information mapped to the principal components of cortical activity even when cortex→SPN connectivity is sparse and different cortical neurons project to each SPN. So, provided that the cortical information encoded in striosome- and matrix-projecting regions is somewhat similar, the decision-dimensions formed by the population should, on average, be similar. To speculate on the function role of the different connectivity, it could be that the regions that are more mSPNsprojecting relay more detailed representations of information, while the regions that are more sSPNs-projecting relay information about how many decision-dimensions to use (a similar effect to the b_{sSPN} term in eq. (1)). This hypothesis might align with functional differences in the regions that project mostly to striosomes versus matrix. For instance, the prefrontal cortex, associated with complex cognitive function, projects mostly to striosomes and inhibits striosomes during conflict[9], perhaps suggesting a functional role in increasing the dimensionality of the decisionspace based on internal representations of task difficulty. Similarly, the orbitofrontal

cortex, associated with high-level decision-making schema[103] and hyperactive during anxiety, projects mostly to striosomes and may communicate contextual information related to the decision.

Supplementary Note 4: Modulation of SPNs by dopamine during the decision to form decisionspace.

We make several hypotheses about dopamine to form our model. First, we hypothesize that dopamine plays a role in modulating SPN activity during decision-making. This follows based on the existing work linking dopamine to second-by-second modulation of SPN firing[104] including work that demonstrates an opposite effect in striosome versus matrix[93]. A recent experiment demonstrated that midbrain dopamine neurons selectively modulate a subpopulation of SPNs[41], perhaps corresponding to a decision-dimension (see **Supplementary Table 6**). Second, we hypothesize that this modulatory effect occurs variably over time and is most pronounced when new important cues appear. This aligns with the literature on RPE[105]. Third, we hypothesize that dopamine modulation should only occur in complex tasks (that require one or more decisiondimension), compatible with evidence that suggests minimal effect of dopamine release on SPNs in simple tasks[41]. Fourth, we hypothesize that dopamine plays an important role in largescale sSPN modulation of mSPN compared to including collaterals, interneurons (choline acetyltransferase interneurons, somatostatin expressing interneurons, and parvalbumin-positive interneurons cross compartment boundaries but likely have primarily local effects[106]), or other possible connections through other brain regions.

Supplementary Note 5: Situations in which different types of decision-spaces form.

In **Figs. 3, 4** and **Supplementary Figs. 3, 4,** we model cases where high- or low-dimensional decision-spaces form in the literature. For instance, in the model in **Fig. 3a-c** of the experimental data plotted in **Supplementary Fig. 3a**, we show that in a T-maze task, a higher-dimensional decision-space tends to form in more “difficult” tasks where there are more environmental dimensions (reward and cost rather than reward or cost separately). In the model in **Supplementary Fig. 3s** of the experimental data in **Supplementary Fig. 3r**, we show that the

decision-space increased over the span of learning. Then we show that the decision-space likely decreases in disorders (**Fig. 4**), perhaps because a low-dimensional decision-space is optimal under disorder conditions (**Supplementary Fig. 6**). Additionally, in **Fig. 5e**, we suggest that a high-dimensional decision-space will form in a high-motivation scenario with high dopamine, and vice versa. So, a high-dimensional decision-space likely forms when 1) there are multiple environmental dimensions, 2) there is reward/cost conflict, 3) after learning, 4) in control animals but not during stress, aging, and Huntington's disease, and 5) when an animal is highly motivated.

When else might a high-dimensional decision-space form? Future work might uncover a single metric predicting the dimension of the decision-space, drawing on the factors we identify and also others. We might expect the urgency with which a decision must be made, for example, to inversely correlate with the dimension of the decision-space. Relying on a single decisiondimension, for instance, might be beneficial in a situation where information along the various decision-dimensions has only been collected for a short period of time, increasing uncertainty (similar to the noise in **Supplementary Fig. 5**). We could also speculate that very late in learning, once a decision becomes routine, the dimension of the decision-space might reduce as unneeded decision-dimensions are deprioritized. Finally, we might expect volatile environments, due to a reduction in signal to noise ratio in information from the cortex, to reduce the dimension of the decision-space for the theoretical reasons we analyze in **Supplementary Fig. 5**.

Supplementary Note 6: Effect of GPi, LHb, and RMTg, and GPe on the direct and indirect pathway spaces.

While recent evidence suggests that there are three pathways from striosomes to daSNC, two direct pathways and one indirect pathway via GPe[23], it remains unresolved whether GPe projects to all daSNC neurons, or only a subset. In the connectivity diagram in **Fig. 1b**, we assume that GPe only projects to a subset of daSNC neurons. Under this assumption, GPi, LHb, and RMTg activity affect both the direct and indirect pathway space (**Fig. 2e**), and GPe affects only the indirect pathway space (**Fig. 2f**). Future studies may lead to confirmation or revision of this assumption. In the case of revision, the mechanics of the model of certain circuit elements would be affected, but not the formation of the direct and indirect pathway decision-space as in **Fig. 5b,c**. Specifically, if the either of the assumptions do not hold, then GPe would affect both the

direct and indirect pathway spaces, and **Fig. 2f** would apply to the direct pathway as well as the indirect pathway, and eq. (2) would be modified.

It is also possible that only a subset of striosomes project to GPi, or that RMTg inhibits only a subset of daSNC neurons. In these case, there would be nonlinear effects of striosome activity on the decision-space, and eq. (2) would be rewritten to reflect selective RMTg influence on certain daSNC subpopulations over others. **Fig. 2e,f** would be revised. However, the effect of dopamine on the direct and indirect pathway spaces in **Fig. 5b,c** would be preserved.

In summary, future modification of the model in the sSPN/GPi/LHb/RMTg/GPe sub-circuits (or introduction of other brain regions, e.g. SNR) might add detail to the process by which these brain regions bias the decision-space. However, any such changes would be self-contained to the circuit prior to the formation of the decision-space, and would not affect, either conceptually or computationally, the influence that daSNC activity has on the formation of the direct and indirect pathway decision-spaces.

Supplementary Note 7: Implications of GPe pathways.

In future computational and experimental work, it will be crucial to examine the GPe neuronal subtypes to understand the mechanisms by which the indirect pathway decision-space forms. GPe neurons can be categorized as prototypical or arkypallidal[107]. The interplay between these neurons has been suggested to enable more nuanced control over action selection[108] by allowing for actions to be restricted or halted entirely when necessary[109,110]. Arkypallidal neurons increase their activity with movement initiation and otherwise have a low basal firing rate especially when compared to prototypical neurons[111,112]. Functionally, Arkypallidal neurons are thought to be capable of canceling an action during initiation through increased activity and GABAergic feedback to the striatum, a mechanism for responding to rapid drastic changes in the environment[107]. Further, Arkypallidal neurons may synergistically activate to modulate proper movement initiation when an action is not being canceled[107], and recent experimental evidence demonstrated a regulatory role in habitual seeking behavior[113]. Prototypical neurons are thought to play a role in information processing and suppression of actions while potentially modulating the reactivity of Arkypallidal neurons[110].

Arkypallidal neurons, but not prototypical GPe neurons, have been found to exert strong influence on the striatum[114], although it is unclear if arkypallidal neurons preferentially target striosomes or matrix. This question will have important implications for future improvements to our model. If arkypallidal neurons are especially striosome-projecting, then our model would suggest that arkypallidal-striosome interplay helps to control the indirect pathway decision-space. This might function similarly to the bi-directional relationship of striosomes and daSNC which we model in **Fig. 5**. However, this arkypallidal-striosome control system would be indirect pathway specific. Alternatively, if it is found that arkypallidal neurons project to matrix preferentially, or to striosomes and matrix roughly evenly, then perhaps arkypallidal neurons function as a switch that can suddenly downshift the decision-space from high-dimensional to low-dimensional if newly arriving information necessitates it. In this second case, arkypallidal neurons would have a more direct effect on action values.

Supplementary Note 8: Compatibility of our model with others of the basal ganglia.

Our model is largely compatible with other models of the basal ganglia, but adds important detail about the different roles of striosomes and matrix. For example, a range of other models consider how the direct and indirect pathways of the basal ganglia interact to moderate action selection[115,116]. Our model offers a simple explanation for several experimental phenomena that are used to fit other models by differentiating the roles of striosomes versus matrix (**Supplementary Fig. 7**). In particular, direct/indirect pathway models more commonly fit data from simple reward and/or cost tasks rather than highly difficult tasks, but our model allows for scaling to difficult tasks with many informational dimensions. Another class of model examines how RPE signals facilitate adaptability[117–119]. Our model does not preclude the possibility that SPNs and/or dopamine may encode an algorithm similar to those used in reinforcement learning but suggests that the primary role of dopamine is to form the decision-space. Our model can additionally be used to expand on other models that explore the role of basal ganglia pathways in performing dimensionality reduction[120], responding to events as sequences that influence each successive action[121,122], or functioning as weights that bias output signals used downstream[123]. In the case of dimensionality reduction models, our model links the process of reducing cortical information directly to decisions. In the case of models of sequential actions, our model forms a

basis for how the striatum can dynamically respond to changes in context over time while also retaining consistency in the decision-space, allowing for chains of motor programs to function with similar rules. In the case of models that examine how the basal ganglia biases outputs, our model specifies how these biases shift from context to context.

Supplementary Note 9: Different roles of striosome versus matrix in reinforcement learning due to opposite plasticity mechanisms.

Our model's inclusion of both striosomes and matrix has important implications for reinforcement learning models of the basal ganglia. In some reinforcement learning models of the circuit, midbrain dopaminergic neurons communicate reward prediction error to SPNs[117,124] similar to the update signal in the classical Q-learning algorithm[125]. After dopamine is released, it affects the plasticity of SPNs. Our model can be used in conjunction with this learning model, if it assumed that learning updates occur after the decision-space forms. Matrix and striosomes have opposite short-term plasticity rules[93]. So, it could be that dopamine induces opposite long-term plasticity changes, although future experimental work would need to verify this. If this hypothesis is demonstrated to be true, then dopamine release might serve a dual purpose in the context of reinforcement learning. Like in the classical view, dopamine would update the policy of possible actions in mSPNs (like an "actor" in an actor-critic algorithm). However, simultaneously, dopamine would update a different function in striosomes, which tightly control the midbrain dopamine neurons that signal the state value function (like the "critic"). So, through the lens of classical reinforcement learning models of the circuit, the different plasticity rules between matrix and striosomes would be functionally convenient.

Supplementary Note 10: Experimental work that informs our model of fast-spiking interneurons (FSIs).

Our model incorporates FSIs by drawing upon key experimental work that characterizes their functional properties and connectivity within the striatal circuitry. FSIs receive excitatory inputs from many cortical neurons[126,127], which themselves exhibit a wide range of firing rates[126]. The cortical to FSI connection is excitatory[128] and current work shows that FSI activity scales linearly

with cortical activity[10,129]. In turn, FSIs exert a well-established inhibitory influence on spiny projection neurons[130]. While the inhibitory nature of this interaction is clear, the precise algebraic operator that best represents this FSI-to-SPN inhibition is less definitive. Experimental evidence can be used to support either a model where it functions as a subtraction or a division[131,132]. Also see **Reasoning behind the FSI model.**

Supplementary Note 11: Rationale for modeling choices and potential alternatives.

In developing our computational model, we evaluated various alternative modeling techniques, such as neural networks, Hidden Markov Models, Bayesian methods, State Space Models, biophysical models, and drift-diffusion models, among others. This note outlines the justifications for our selected modeling approaches and considers other methods that could yield comparable results.

Our model represents the decision-making axes (decision-dimensions) as the principal components of cortical data. This choice directly links the circuit's processes to common data science operations. While this decision-space model could be conceptually preserved by substituting principal components with, for instance, the independent components[133] of cortical data, such modifications might compromise computational convenience.

To derive choice and deliberation time for an arbitrary number of potential actions, we aimed for a framework analogous to drift-diffusion models, which have proven successful in other Basal Ganglia research[134]. However, classical drift-diffusion models are typically limited to two outcomes, though multi-outcome extensions exist[135]. Our model was designed to accommodate an unlimited number of possible actions. It successfully reproduces choice behavior, as demonstrated in the T-maze task (**Supplementary Fig. 3b,c**). While interacting population models[136] could also achieve our goal, our primary focus was on choice outcomes (i.e., the choice itself, deliberation time, and its distribution) rather than the specifics of neural recruitment. Alternative approaches, such as treating choice as a Bayesian process emerging from circuit activity[137] or employing an intermediate layer like in a Hidden Markov Model[138], might also be viable, although deriving deliberation time is less common with these model classes.

For modeling physiological connections, we used relative firing rates to represent the overall activities of circuit elements. This approach effectively linked the macroscopic activities of different brain regions within the circuit. Although models that incorporate the precise timing of individual neuron spikes have been used to describe connections in specific parts of the circuit with high fidelity[139,140], the conceptual framework of the decision-space model does not necessitate this level of granularity.

Supplementary Note 12: Influence of cost and reward on LHb, RMTg, and daSNC activity.

Our model captures how varying cost levels modulate LHb and RMTg activities. These regions have been found to be highly sensitive to cost levels[21,34,141–143]. LHb and RMTg exhibit a roughly negative linear response to changes in reward level and a positive linear response to changes in cost level[141,144]. RMTg projects to daSNC via strong inhibition (**Figure 1, Supplementary Table 1**), where the mean activity of a daSNC subpopulation typically responds positively to increases in reward and negatively to decreases in reward[141,144].

Instructions to run code.

For code, see https://github.com/dirkbeck/DM_space_model or the release at https://zenodo.org/records/15433189.

The subdirectories correspond to different topics in the manuscript. Functions used across the topics sit outside the subdirectories. There is also an “Original Functions” folder for preprocessing experimental data.

The topics correspond to the latest version of the manuscript (updated after one round of review) as follows:

- model_overview: **Figs. 1,2** and **Supplementary Fig. 2**
- model_tests: **Fig. 3a-d** and **Supplementary Fig. 3a,o-x**
- disorder_hypotheses: **Fig. 4** and **Supplementary Fig. 4m-r**
- dynamic_model_and_neural_net: **Fig. 5** and **Supplementary Figs. 5, 6, 7a-i**
- importance_and_RPEs: **Supplementary Fig. 7k,l.**
- day_to_day_space_sampling: **Fig. 6** and **Supplementary Fig. 8**
- circuit_trajectories: **Fig. 7**, **Supplementary Fig. 9**
- neuron_pair_analysis: **Fig. 3f**, **Supplementary Fig. 3k-p**
- Cross Correlation Pattern Counts: **Fig. 3e**, **Supplementary Fig. 3b-n**, **Supplementary Fig. 3a-j**.

For additional detail on directory<->figure mapping, please refer to the Methods section of the manuscript, where the analysis corresponding to each figure is explained and a link is provided to the github.

All .m files run in MATLAB R2021a. To run the code:

1) Git clone the repository onto a local machine.
2) Run the file of interest in MATLAB. Note that (except for the exceptions mentioned below) the codes either A) do not require functions in other files, or B) require functions in other files that are automatically added to the path when the file is run.

Fig. 2

- **Fig. 2a,b**: model_overview/cartoon_of_striosome_matrix_roles.m - **Fig. 2c,d**: model_overview/sSPN_versus_mSPN_effect_on_decision_space_and_action_values.m
- **Fig. 2e,f**: model_overview/GPi_LHb_RMTg_DA_model.m

Supplementary Fig. 2

- **Supplementary Fig. 2a**: model_overview/ctx_FSI_SPN_firing_rates.m

- **Supplementary Fig. 2b**: model_overview/GPi_LH_RMTg_DA_operation_cartoons.m
- **Supplementary Fig. 2c,d**: model_overview/GPi_model.m
- **Supplementary Fig. 2e,f**: model_overview/LHb_model.m
- **Supplementary Fig. 2g-i**: model_overview/GPi_LHb_RMTg_DA_model.m
- **Supplementary Fig. 2k,**l: model_overview/mSPN_multinomial_regression_example
- **Supplementary Fig. 2m,n**: model_overview/weiner_process_illustration.m
- **Supplementary Fig. 2o,p**: model_overview/weiner_process_illustration.m
- **Supplementary Fig. 2q-r**: model_overview/dimensionality_from_SPN_activity

Fig. 3

- **Fig. 3a,b**. model_tests/friedman2015optogeneticmanipulation.m - **Fig. 3d**. model_tests/ctx_sSPN_mSPN_coordinated_activity.n - **Fig. 3e**. See Exception 2. - **Fig. 3f**. See Exception 1.

Supplementary Fig. 3

- **Supplementary Fig. 3a.** model_tests/friedman2015optogeneticmanipulation.m - **Supplementary Fig. 3b-d**. See Exception 2.
- **Supplementary Fig. 3e,f.** See Exception 1.
- **Supplementary Fig. 3g**. See Exception 2.
- **Supplementary Fig. 3h-n.** See Exception 2.
- **Supplementary Fig. 3o-x**. model_tests/comparison_to_benchmarks.m

Fig. 4

- **Fig. 4a.** disorder_hypotheses/Friedman2017summary.m
- **Fig. 4b,c.** disorder_hypotheses/Friedman2017_lowD_space.m
- **Fig. 4d,e.** disorder_hypotheses/altered_choice_after_space_transition.m
- **Fig. 4f.** disorder_hypotheses/space_dimensionality_vs_FSI.m
- **Fig. 4h.** disorder_hypotheses/Friedman2020_lowD_space.m

Supplementary Fig. 4

- **Supplementary Fig. 4a-j**
- **Supplementary Fig. 4k,l.** See Exception 1.
- **Supplementary Fig. 4m,n,q,r.** disorder_hypotheses/ctx_to_FSI_synchrony_analysis.m - **Supplementary Fig. 4o,p.** See Exception 1.

Fig. 5

- **Fig. 5d-i.** dynamic_model_and_neural_net/sSPN_DA_mSPN_dynamic_interaction.m

Supplementary Fig. 5

- **Supplementary Fig. 5b-e.** dynamic_model_and_neural_net/neural_network_model.m - **Supplementary Fig. 5g.** dynamic_model_and_neural_net/dimension_discrimination_vs_sparsity.m

Fig. 6

- **Fig. 6a.** sampling_space_based_on_activity/decision_space_sampling.py
- **Fig. 6b,c.** sampling_space_based_on_activity/subjective_value_scores_by_space.m
- **Fig. 6e.** sampling_space_based_on_activity/subjective_value_score_extremes.m

Supplementary Fig. 6

- dynamic_model_and_neural_net/cortical_snr.m

Fig. 7

- **Fig. 7b-e.** circuit_trajectories/advantage_cost_net_advantage_example.m
- **Fig. 7f,g.** circuit_trajectories/advantage_cost_net_advantage_example.m

Supplementary Fig. 7

- **Supplementary Fig. 7c-f.** dynamic_model_and_neural_net/direct_vs_indirect_pathway_SV.m - **Supplementary Fig. 7i.** dynamic_model_and_neural_net/direct_vs_indirect_pathway_proximity_theory.m

Supplementary Fig. 8

- **Supplementary Fig. 8d.** sampling_space_based_on_activity/subjective_value_scores_by_space.m - **Supplementary Fig. 8f.** sampling_space_based_on_activity/subjective_value_score_extremes.m

Supplementary Fig. 9

- **Fig. 9a.** circuit_trajectories/sSNC_DA_LH_trajectories_examples2.m
- **Fig. 9b,c.** circuit_trajectories/changing_space_value_between_trials.m

Exception 1. See neuron_pair_analysis/run_me.m for instructions.

Exception 2. See Cross Correlation Pattern Counts/createPaperFigures.m for instructions.

Key resources table.

REAGENT or RESOURCE	SOURCE	IDENTIFIER

Deposited data		
Corticostriosomal Circuit Stress Experiment	Friedman et al. (2017)	https://data.mendeley.com/datasets/z9jd8xhj84/1
A decision-space model explains contextspecific decision-making	This paper	https://doi.org/10.7910/DVN/SMKW0I
Overview of the model	This paper	https://github.com/dirkbeck/DM_space_model/tree/main/model_overview
Tests of the model	This paper	https://github.com/dirkbeck/DM_space_model/tree/main/model_tests
Disorder hypotheses	This paper	https://github.com/dirkbeck/DM_space_model/tree/main/disorder_hypotheses
Instances 2 (sparse connectivity) and 3 (dynamics)	This paper	https://github.com/dirkbeck/DM_space_model/tree/main/dynamic_model_and_neural_net
Decision-space shifts in a T-maze learning task	This paper	https://github.com/dirkbeck/DM_space_model/tree/main/importance_and_RPEs
Day to day differences and disorder comorbidity	This paper	https://github.com/dirkbeck/DM_space_model/tree/main/day_to_day_space_sampling
Circuit adjustment between trials	This paper	https://github.com/dirkbeck/DM_space_model/tree/main/circuit_trajectories

Analysis of the correlation between cortical neurons, FSIs, sSPNs, and mSPNs during decision-making	This paper	https://github.com/dirkbeck/DM_space_model/tree/main/neuron_pair_analysis
Analysis of the functional connectivity of sSPNs and mSPNs during decision-making	This paper	https://github.com/dirkbeck/DM_space_model/tree/main/Cross%20Correlation%20Pattern%20Counts
Software and algorithms		
MATLAB R2021a	Mathworks	https://www.mathworks.com/products/matlab.html

Supplemental References

1. Reiner, A. Corticostriatal projection neurons – dichotomous types and dichotomous functions. *Front. Neuroanat.* **4**, (2010).
2. Lanciego, J. L., Luquin, N. & Obeso, J. A. Functional neuroanatomy of the basal ganglia. *Cold Spring Harb Perspect Med* **2**, a009621 (2012).
3. Graybiel, A. M. & Matsushima, A. Striosomes and Matrisomes: Scaffolds for Dynamic Coupling of Volition and Action. *Annu. Rev. Neurosci.* **46**, 359–380 (2023).
4. Miyamoto, Y., Katayama, S., Shigematsu, N., Nishi, A. & Fukuda, T. Striosome-based map of the mouse striatum that is conformable to both cortical afferent topography and uneven distributions of dopamine D1 and D2 receptor-expressing cells. *Brain structure & function* **223**, 4275–4291 (2018).
5. Martel, A.-C. & Galvan, A. Connectivity of the corticostriatal and thalamostriatal systems in normal and parkinsonian states: An update. *Neurobiology of Disease* **174**, 105878 (2022).

6. Crittenden, J. R. & Graybiel, A. M. Basal Ganglia disorders associated with imbalances in the striatal striosome and matrix compartments. *Front Neuroanat* **5**, 59 (2011).
7. Eblen, F. & Graybiel, A. M. Highly restricted origin of prefrontal cortical inputs to striosomes in the macaque monkey. *J Neurosci* **15**, 5999–6013 (1995).
8. McGregor, M. M. *et al.* Functionally Distinct Connectivity of Developmentally Targeted Striosome Neurons. *Cell Rep* **29**, 1419-1428.e5 (2019).
9. Friedman, A. *et al.* A Corticostriatal Path Targeting Striosomes Controls Decision-Making under Conflict. *Cell* **161**, 1320–1333 (2015).
10. Friedman, A. *et al.* Striosomes Mediate Value-Based Learning Vulnerable in Age and Huntington's Model. *Cell* **183**, 918-934.e49 (2020).
11. Friedman, A. *et al.* Chronic Stress Alters Striosome-Circuit Dynamics, Leading to Aberrant Decision-Making. *Cell* **171**, 1191-1205.e28 (2017).
12. Fujiyama, F. *et al.* Exclusive and common targets of neostriatofugal projections of rat striosome neurons: a single neuron-tracing study using a viral vector. *Eur J Neurosci* **33**, 668–677 (2011).
13. Gerfen, C. R. The neostriatal mosaic: compartmentalization of corticostriatal input and striatonigral output systems. *Nature* **311**, 461–4 (1984).
14. Smith, J. B. *et al.* Genetic-Based Dissection Unveils the Inputs and Outputs of Striatal Patch and Matrix Compartments. *Neuron* **91**, 1069–1084 (2016).
15. Rajakumar, N., Elisevich, K. & Flumerfelt, B. A. Compartmental origin of the striatoentopeduncular projection in the rat. *J Comp Neurol* **331**, 286–296 (1993).
16. Hong, S. & Hikosaka, O. Diverse sources of reward value signals in the basal ganglia nuclei transmitted to the lateral habenula in the monkey. *Front Hum Neurosci* **7**, 778 (2013).
17. Hong, S. & Hikosaka, O. The Globus Pallidus Sends Reward-Related Signals to the Lateral Habenula. *Neuron* **60**, 720–729 (2008).

18. Hong, S. *et al.* Predominant Striatal Input to the Lateral Habenula in Macaques Comes from Striosomes. *Curr Biol* **29**, 51-61.e5 (2019).

19. Weglage, M. *et al. Sst+ GPi Output Neurons Provide Direct Feedback to Key Nodes of the Basal Ganglia and Drive Behavioral Flexibility*. http://biorxiv.org/lookup/doi/10.1101/2022.03.16.484460 (2022) doi:10.1101/2022.03.16.484460.

20. Gonçalves, L., Sego, C. & Metzger, M. Differential projections from the lateral habenula to the rostromedial tegmental nucleus and ventral tegmental area in the rat. *J of Comparative Neurology* **520**, 1278–1300 (2012).

21. Jhou, T. C., Fields, H. L., Baxter, M. G., Saper, C. B. & Holland, P. C. The Rostromedial Tegmental Nucleus (RMTg), a GABAergic Afferent to Midbrain Dopamine Neurons, Encodes Aversive Stimuli and Inhibits Motor Responses. *Neuron* **61**, 786–800 (2009).

22. Smith, R. J., Vento, P. J., Chao, Y. S., Good, C. H. & Jhou, T. C. Gene expression and neurochemical characterization of the rostromedial tegmental nucleus (RMTg) in rats and mice. *Brain Struct Funct* **224**, 219–238 (2019).

23. Lazaridis, I. *et al.* Striosomes control dopamine via dual pathways paralleling canonical basal ganglia circuits. *Current Biology* S0960982224013381 (2024) doi:10.1016/j.cub.2024.09.070.

24. Jaquins-Gerstl, A., Nesbitt, K. M. & Michael, A. C. In vivo evidence for the unique kinetics of evoked dopamine release in the patch and matrix compartments of the striatum. *Anal Bioanal Chem* (2021) doi:10.1007/s00216-021-03300-z.

25. Brimblecombe, K. R. & Cragg, S. J. The Striosome and Matrix Compartments of the Striatum: A Path through the Labyrinth from Neurochemistry toward Function. *ACS Chem Neurosci* **8**, 235–242 (2017).

26. Salinas, A. G., Davis, M. I., Lovinger, D. M. & Mateo, Y. Dopamine dynamics and cocaine sensitivity differ between striosome and matrix compartments of the striatum.

Neuropharmacology **108**, 275–283 (2016).

27. Bloem, B. *et al.* Multiplexed action-outcome representation by striatal striosome-matrix compartments detected with a mouse cost-benefit foraging task. *Nat Commun* **13**, 1541 (2022).

28. Xiao, X. *et al.* A Genetically Defined Compartmentalized Striatal Direct Pathway for Negative Reinforcement. *Cell* **183**, 211-227.e20 (2020).

29. Weglage, M. *et al.* Complete representation of action space and value in all dorsal striatal pathways. *Cell Rep* **36**, 109437 (2021).

30. Münte, T. F. *et al.* The human globus pallidus internus is sensitive to rewards – Evidence from intracerebral recordings. *Brain Stimulation* **10**, 657–663 (2017).

31. Stephenson-Jones, M. *et al.* A basal ganglia circuit for evaluating action outcomes. *Nature* **539**, 289–293 (2016).

32. Matsumoto, M. & Hikosaka, O. Lateral habenula as a source of negative reward signals in dopamine neurons. *Nature* **447**, 1111-U11 (2007).

33. Lee, H. & Hikosaka, O. Lateral habenula neurons signal step-by-step changes of reward prediction. *iScience* **25**, 105440 (2022).

34. Stopper, C. M. & Floresco, S. B. What's better for me? Fundamental role for lateral habenula in promoting subjective decision biases. *Nat Neurosci* **17**, 33–35 (2014).

35. Vento, P. J., Burnham, N. W., Rowley, C. S. & Jhou, T. C. Learning From One's Mistakes: A Dual Role for the Rostromedial Tegmental Nucleus in the Encoding and Expression of Punished Reward Seeking. *Biological Psychiatry* **81**, 1041–1049 (2017).

36. Fiorillo, C. D., Tobler, P. N. & Schultz, W. Discrete Coding of Reward Probability and Uncertainty by Dopamine Neurons. *Science* **299**, 1898–1902 (2003).

37. Matsumoto, M. & Hikosaka, O. Two types of dopamine neuron distinctly convey positive and negative motivational signals. *Nature* **459**, 837–841 (2009).

38. Gan, J. O., Walton, M. E. & Phillips, P. E. M. Dissociable cost and benefit encoding of future rewards by mesolimbic dopamine. *Nat Neurosci* **13**, 25–27 (2010).

39. Bromberg-Martin, E. S., Matsumoto, M. & Hikosaka, O. Dopamine in Motivational Control: Rewarding, Aversive, and Alerting. *Neuron* **68**, 815–834 (2010).

40. Kim, H. R. *et al.* A Unified Framework for Dopamine Signals across Timescales. *Cell* **183**, 1600-1616.e25 (2020).

41. Long, C. *et al.* Constraints on the subsecond modulation of striatal dynamics by physiological dopamine signaling. *Nat Neurosci* **27**, 1977–1986 (2024).

42. Samejima, K., Ueda, Y., Doya, K. & Kimura, M. Representation of action-specific reward values in the striatum. *Science* **310**, 1337–40 (2005).

43. Seo, M., Lee, E. & Averbeck, B. B. Action Selection and Action Value in Frontal-Striatal Circuits. *Neuron* **74**, 947–960 (2012).

44. Parker, J. G. *et al.* Diametric neural ensemble dynamics in parkinsonian and dyskinetic states. *Nature* **557**, 177–182 (2018).

45. Peak, J., Chieng, B., Hart, G. & Balleine, B. W. Striatal direct and indirect pathway neurons differentially control the encoding and updating of goal-directed learning. *eLife* **9**, e58544 (2020).

46. Maltese, M., March, J. R., Bashaw, A. G. & Tritsch, N. X. Dopamine differentially modulates the size of projection neuron ensembles in the intact and dopamine-depleted striatum. *Elife* **10**, e68041 (2021).

47. Barbera, G. *et al.* Spatially Compact Neural Clusters in the Dorsal Striatum Encode Locomotion Relevant Information. *Neuron* **92**, 202–213 (2016).

48. Yoshizawa, T., Ito, M. & Doya, K. Reward-predictive neural activities in striatal striosome compartments. *eNeuro* **5:e0367**, (2018).

49. Nadel, J. A., Pawelko, S. S., Copes-Finke, D., Neidhart, M. & Howard, C. D. Lesion of striatal patches disrupts habitual behaviors and increases behavioral variability. *PLoS One* **15**, e0224715 (2020).

50. Beste, C., Moll, C. K. E., Pötter-Nerger, M. & Münchau, A. Striatal Microstructure and Its Relevance for Cognitive Control. *Trends Cogn Sci* **22**, 747–751 (2018).

51. Lawhorn, C., Smith, D. M. & Brown, L. L. Partial ablation of mu-opioid receptor rich striosomes produces deficits on a motor-skill learning task. *Neuroscience* **163**, 109–119 (2009).

52. Corbit, V. L., Ahmari, S. E. & Gittis, A. H. A Corticostriatal Balancing Act Supports Skill Learning. *Neuron* **96**, 253–255 (2017).

53. Grillner, S. & Robertson, B. The basal ganglia downstream control of brainstem motor centres—an evolutionarily conserved strategy. *Current Opinion in Neurobiology* **33**, 47–52 (2015).

54. Balleine, B. W., Delgado, M. R. & Hikosaka, O. The Role of the Dorsal Striatum in Reward and Decision-Making. 5.

55. Lazaridis, I. *et al.* Striosomes Target Nigral Dopamine-Containing Neurons via Direct-D1 and Indirect-D2 Pathways Paralleling Classic Direct-Indirect Basal Ganglia Systems. Preprint at https://doi.org/10.1101/2024.06.01.596922 (2024).

56. Fujiyama, F., Karube, F. & Hirai, Y. Globus pallidus is not independent from striatal direct pathway neurons: an up-to-date review. *Mol Brain* **17**, 34 (2024).

57. Cox, S. M. L. *et al.* Striatal D1 and D2 signaling differentially predict learning from positive and negative outcomes. *Neuroimage* **109**, 95–101 (2015).

58. Hikosaka, O. *et al.* Direct and indirect pathways for choosing objects and actions. *Eur J Neurosci* **49**, 637–645 (2019).

59. Harris, K. D. & Thiele, A. Cortical state and attention. *Nat Rev Neurosci* **12**, 509–523

(2011).

60. O'Doherty, J. P. Contributions of the ventromedial prefrontal cortex to goal-directed action selection. *Annals of the New York Academy of Sciences* **1239**, 118–129 (2011).

61. Ostlund, S. B. & Balleine, B. W. The Contribution of Orbitofrontal Cortex to Action Selection. *Annals of the New York Academy of Sciences* **1121**, 174–192 (2007).

62. Sharpe, M. J. *et al.* An Integrated Model of Action Selection: Distinct Modes of Cortical Control of Striatal Decision Making. *Annu. Rev. Psychol.* **70**, 53–76 (2019).

63. Kim, H. F. & Hikosaka, O. Distinct Basal Ganglia Circuits Controlling Behaviors Guided by Flexible and Stable Values. *Neuron* **79**, 1001–1010 (2013).

64. Lee, S., Yu, L. Q., Lerman, C. & Kable, J. W. Subjective value, not a gridlike code, describes neural activity in ventromedial prefrontal cortex during value-based decisionmaking. *NeuroImage* **237**, 118159 (2021).

65. Padoa-Schioppa, C. & Cai, X. The orbitofrontal cortex and the computation of subjective value: consolidated concepts and new perspectives. *Annals of the New York Academy of Sciences* **1239**, 130–137 (2011).

66. Viviani, R. Neural Correlates of Emotion Regulation in the Ventral Prefrontal Cortex and the Encoding of Subjective Value and Economic Utility. *Front. Psychiatry* **5**, (2014).

67. Hikida, T., Kimura, K., Wada, N., Funabiki, K. & Nakanishi, S. Distinct Roles of Synaptic Transmission in Direct and Indirect Striatal Pathways to Reward and Aversive Behavior. *Neuron* **66**, 896–907 (2010).

68. Stephenson-Jones, M. Pallidal circuits for aversive motivation and learning. *Current Opinion in Behavioral Sciences* **26**, 82–89 (2019).

69. Brown, J., Bullock, D. & Grossberg, S. How the Basal Ganglia Use Parallel Excitatory and Inhibitory Learning Pathways to Selectively Respond to Unexpected Rewarding Cues. *J. Neurosci.* **19**, 10502–10511 (1999).

70. Mollick, J. A. *et al.* The Neural Correlates of Cued Reward Omission. *Front. Hum. Neurosci.* **15**, 615313 (2021).
71. Volkow, N. D. *et al.* Cocaine Cues and Dopamine in Dorsal Striatum: Mechanism of Craving in Cocaine Addiction. *Journal of Neuroscience* **26**, 6583–6588 (2006).
72. Hillman, K. L. & Bilkey, D. K. Neurons in the Rat Anterior Cingulate Cortex Dynamically Encode Cost–Benefit in a Spatial Decision-Making Task. *J. Neurosci.* **30**, 7705–7713 (2010).
73. Rushworth, M. F. S., Noonan, M. P., Boorman, E. D., Walton, M. E. & Behrens, T. E. Frontal cortex and reward-guided learning and decision-making. *Neuron* **70**, 1054–1069 (2011).
74. Howell, N. A. *et al.* Preliminary evidence for human globus pallidus pars interna neurons signaling reward and sensory stimuli. *Neuroscience* **328**, 30–39 (2016).
75. Bromberg-Martin, E. S. & Hikosaka, O. Lateral habenula neurons signal errors in the prediction of reward information. *Nat Neurosci* **14**, 1209–1216 (2011).
76. Bayer, H. M. & Glimcher, P. W. Midbrain dopamine neurons encode a quantitative reward prediction error signal. *Neuron* **47**, 129–41 (2005).
77. Watabe-Uchida, M., Eshel, N. & Uchida, N. Neural Circuitry of Reward Prediction Error. *Annu Rev Neurosci* **40**, 373–394 (2017).
78. Lak, A., Stauffer, W. R. & Schultz, W. Dopamine prediction error responses integrate subjective value from different reward dimensions. *Proceedings of the National Academy of Sciences of the United States of America* **111**, 2343–8 (2014).
79. Aupperle, R. L. & Paulus, M. P. Neural systems underlying approach and avoidance in anxiety disorders. *Dialogues in clinical neuroscience* **12**, 517–31 (2010).
80. Gleichgerrcht, E., Ibanez, A., Roca, M., Torralva, T. & Manes, F. Decision-making cognition in neurodegenerative diseases. *Nature reviews. Neurology* **6**, 611–23 (2010).

81. Shabel, S. J., Proulx, C. D., Piriz, J. & Malinow, R. Mood regulation. GABA/glutamate corelease controls habenula output and is modified by antidepressant treatment. *Science* **345**, 1494–8 (2014).

82. Wang, Y. *et al.* Habenula deep brain stimulation for intractable schizophrenia: a pilot study. *Neurosurgical Focus* **49**, E9 (2020).

83. Gold, P. W. & Kadriu, B. A Major Role for the Lateral Habenula in Depressive Illness: Physiologic and Molecular Mechanisms. *Front Psychiatry* **10**, 320 (2019).

84. Hu, H., Cui, Y. & Yang, Y. Circuits and functions of the lateral habenula in health and in disease. *Nat Rev Neurosci* **21**, 277–295 (2020).

85. Zhang, L. *et al.* Altered Volume and Functional Connectivity of the Habenula in Schizophrenia. *Front. Hum. Neurosci.* **11**, 636 (2017).

86. Hikosaka, O. The habenula: from stress evasion to value-based decision-making. *Nat Rev Neurosci* **11**, 503–513 (2010).

87. Proulx, C. D., Hikosaka, O. & Malinow, R. Reward processing by the lateral habenula in normal and depressive behaviors. *Nat Neurosci* **17**, 1146–1152 (2014).

88. Hikosaka, O., Sesack, S. R., Lecourtier, L. & Shepard, P. D. Habenula: Crossroad between the Basal Ganglia and the Limbic System. *Journal of Neuroscience* **28**, 11825–11829 (2008).

89. Lawhorn, C., Smith, D. M. & Brown, L. L. Striosome-matrix pathology and motor deficits in the YAC128 mouse model of Huntington's disease. *Neurobiology of Disease* **32**, 471–478 (2008).

90. Ferhat, A.-T. *et al.* Excessive self-grooming, gene dysregulation and imbalance between the striosome and matrix compartments in the striatum of Shank3 mutant mice. *Front. Mol. Neurosci.* **16**, 1139118 (2023).

91. Calabresi, P., Picconi, B., Tozzi, A., Ghiglieri, V. & Di Filippo, M. Direct and indirect pathways of basal ganglia: a critical reappraisal. *Nat Neurosci* **17**, 1022–1030 (2014).

92. He, J. *et al.* Transcriptional and anatomical diversity of medium spiny neurons in the primate striatum. *Curr Biol* S0960-9822(21)01369–5 (2021) doi:10.1016/j.cub.2021.10.015.

93. Prager, E. M. *et al.* Dopamine Oppositely Modulates State Transitions in Striosome and Matrix Direct Pathway Striatal Spiny Neurons. *Neuron* **108**, 1091-1102.e5 (2020).

94. Lévesque, M. & Parent, A. The striatofugal fiber system in primates: A reevaluation of its organization based on single-axon tracing studies. *Proc. Natl. Acad. Sci. U.S.A.* **102**, 11888–11893 (2005).

95. Bevan, M. D., Bolam, J. P. & Crossman, A. R. Convergent Synaptic Input From the Neostriatum and the Subthalamus Onto Identified Nigrothalamic Neurons in the Rat. *Eur J of Neuroscience* **6**, 320–334 (1994).

96. Hajós, M. & Greenfield, S. A. Synaptic connections between pars compacta and pars reticulata neurones: electrophysiological evidence for functional modules within the substantia nigra. *Brain Research* **660**, 216–224 (1994).

97. Ragsdale, C. W. & Graybiel, A. M. Fibers from the basolateral nucleus of the amygdala selectively innervate striosomes in the caudate nucleus of the cat. *J of Comparative Neurology* **269**, 506–522 (1988).

98. Fujiyama, F., Unzai, T. & Karube, F. Thalamostriatal projections and striosome-matrix compartments. *Neurochem Int* **125**, 67–73 (2019).

99. Ragsdale, C. W. & Graybiel, A. M. Compartmental organization of the thalamostriatal connection in the cat. *J of Comparative Neurology* **311**, 134–167 (1991).

100. Unzai, T., Kuramoto, E., Kaneko, T. & Fujiyama, F. Quantitative Analyses of the Projection of Individual Neurons from the Midline Thalamic Nuclei to the Striosome and Matrix Compartments of the Rat Striatum. *Cereb Cortex* **27**, 1164–1181 (2017).

101. Martínez-Selva, J. M., Sánchez-Navarro, J. P., Bechara, A. & Román, F. [Brain mechanisms involved in decision-making]. *Rev Neurol* **42**, 411–418 (2006).

102. Bergman, H. *et al.* Physiological aspects of information processing in the basal ganglia of normal and parkinsonian primates. *Trends in Neurosciences* **21**, 32–38 (1998).

103. Zhou, J. *et al.* Evolving schema representations in orbitofrontal ensembles during learning. *Nature* **590**, 606–611 (2021).

104. Hamid, A. A. *et al.* Mesolimbic dopamine signals the value of work. *Nat. Neurosci.* **19**, 117–26 (2016).

105. Schultz, W. Dopamine reward prediction-error signalling: a two-component response. *Nat Rev Neurosci* **17**, 183–195 (2016).

106. Assous, M. & Tepper, J. M. Excitatory extrinsic afferents to striatal interneurons and interactions with striatal microcircuitry. *Eur J of Neuroscience* **49**, 593–603 (2019).

107. Guilhemsang, L. & Mallet, N. P. Arkypallidal neurons in basal ganglia circuits: Unveiling novel pallidostriatal loops? *Current Opinion in Neurobiology* **84**, 102814 (2024).

108. Bogacz, R., Martin Moraud, E., Abdi, A., Magill, P. J. & Baufreton, J. Properties of Neurons in External Globus Pallidus Can Support Optimal Action Selection. *PLoS Comput Biol* **12**, e1005004 (2016).

109. Diesburg, D. A. & Wessel, J. R. The Pause-then-Cancel model of human action-stopping: Theoretical considerations and empirical evidence. *Neuroscience & Biobehavioral Reviews* **129**, 17–34 (2021).

110. Giossi, C., Rubin, J. E., Gittis, A., Verstynen, T. & Vich, C. Rethinking the external globus pallidus and information flow in cortico-basal ganglia-thalamic circuits. *Eur J of Neuroscience* **60**, 6129–6144 (2024).

111. Deffains, M., Iskhakova, L. & Bergman, H. Stop and Think about Basal Ganglia Functional Organization: The Pallido-Striatal “Stop” Route. *Neuron* **89**, 237–239 (2016).

112. Mallet, N. *et al.* Arkypallidal Cells Send a Stop Signal to Striatum. *Neuron* **89**, 308–316 (2016).

113. Baker, M. *et al.* External globus pallidus input to the dorsal striatum regulates habitual seeking behavior in male mice. *Nat Commun* **14**, 4085 (2023).

114. Aristieta, A. *et al.* A Disynaptic Circuit in the Globus Pallidus Controls Locomotion Inhibition. *Current Biology* **31**, 707-721.e7 (2021).

115. Schroll, H. & Hamker, F. H. Computational models of basal-ganglia pathway functions: focus on functional neuroanatomy. *Front. Syst. Neurosci.* **7**, (2013).

116. Gurney, K., Prescott, T. J. & Redgrave, P. A computational model of action selection in the basal ganglia. II. Analysis and simulation of behaviour. *Biol Cybern* **84**, 411–423 (2001).

117. Möller, M., Manohar, S. & Bogacz, R. Uncertainty–guided learning with scaled prediction errors in the basal ganglia. *PLoS Comput Biol* **18**, e1009816 (2022).

118. Morita, K. & Kato, A. Striatal dopamine ramping may indicate flexible reinforcement learning with forgetting in the cortico-basal ganglia circuits. *Front. Neural Circuits* **8**, (2014).

119. Dabney, W. *et al.* A distributional code for value in dopamine-based reinforcement learning. *Nature* **577**, 671–675 (2020).

120. Bar-Gad, I., Morris, G. & Bergman, H. Information processing, dimensionality reduction and reinforcement learning in the basal ganglia. *Progress in Neurobiology* **71**, 439–473 (2003).

121. Berns, G. S. & Sejnowski, T. J. A Computational Model of How the Basal Ganglia Produce Sequences. *Journal of Cognitive Neuroscience* **10**, 108–121 (1998).

122. Garr, E. Contributions of the basal ganglia to action sequence learning and performance. *Neuroscience & Biobehavioral Reviews* **107**, 279–295 (2019).

123. Humphries, M. D. The computational bottleneck of basal ganglia output (and what to do about it). Preprint at https://doi.org/10.1101/2024.10.23.619790 (2024).

124. Mikhael, J. G. & Bogacz, R. Learning Reward Uncertainty in the Basal Ganglia. *PLoS Comput Biol* **12**, e1005062 (2016).

125. Watkins, C. Learning From Delayed Rewards. (1989).

126. Berke, J. D. Functional properties of striatal fast-spiking interneurons. *Frontiers in systems neuroscience* **5**, 45 (2011).

127. McKeon, P. N., Bunce, G. W., Patton, M. H., Chen, R. & Mathur, B. N. Cortical control of striatal fast-spiking interneuron synchrony. *The Journal of Physiology* **600**, 2189–2202 (2022).

128. Sciamanna, G., Ponterio, G., Mandolesi, G., Bonsi, P. & Pisani, A. Optogenetic stimulation reveals distinct modulatory properties of thalamostriatal vs corticostriatal glutamatergic inputs to fast-spiking interneurons. *Sci Rep* **5**, 16742 (2015).

129. Peters, A. J., Fabre, J. M. J., Steinmetz, N. A., Harris, K. D. & Carandini, M. Striatal activity topographically reflects cortical activity. *Nature* **591**, 420–425 (2021).

130. Plenz, D. & Kitai, S. T. Up and Down States in Striatal Medium Spiny Neurons Simultaneously Recorded with Spontaneous Activity in Fast-Spiking Interneurons Studied in Cortex–Striatum–Substantia Nigra Organotypic Cultures. *J. Neurosci.* **18**, 266–283 (1998).

131. Humphries, M. D., Wood, R. & Gurney, K. Reconstructing the Three-Dimensional GABAergic Microcircuit of the Striatum. *PLoS Comput Biol* **6**, e1001011 (2010).

132. Liénard, J. & Girard, B. A biologically constrained model of the whole basal ganglia addressing the paradoxes of connections and selection. *J Comput Neurosci* **36**, 445–468 (2014).

133. Hyvärinen, A. Independent component analysis: recent advances. *Phil. Trans. R. Soc. A.* **371**, 20110534 (2013).

134. Gupta, A. *et al.* Neural Substrates of the Drift-Diffusion Model in Brain Disorders. *Front. Comput. Neurosci.* **15**, 678232 (2022).

135. Roxin, A. Drift–diffusion models for multiple-alternative forced-choice decision making. *J. Math. Neurosc.* **9**, 5 (2019).

136. Murray, J. D. Models for Interacting Populations. in *Mathematical Biology* (ed. Murray, J. D.) vol. 17 79–118 (Springer New York, New York, NY, 1993).

137. Wager, T. D. *et al.* A Bayesian Model of Category-Specific Emotional Brain Responses. *PLoS Comput Biol* **11**, e1004066 (2015).

138. Friedman, A., Keselman, M. D., Gibb, L. G. & Graybiel, A. M. A multistage mathematical approach to automated clustering of high-dimensional noisy data. *Proc Natl Acad Sci U S A* **112**, 4477–4482 (2015).

139. Abeles, M., Hayon, G. & Lehmann, D. Modeling Compositionality by Dynamic Binding of Synfire Chains. *J Comput Neurosci* **17**, 179–201 (2004).

140. Hayon, G., Abeles, M. & Lehmann, D. A Model for Representing the Dynamics of a System of Synfire Chains. *J Comput Neurosci* **18**, 41–53 (2005).

141. Jhou, T. C. The rostromedial tegmental (RMTg) “brake” on dopamine and behavior: A decade of progress but also much unfinished work. *Neuropharmacology* **198**, 108763 (2021).

142. Baker, P. M., Rao, Y., Rivera, Z. M. G., Garcia, E. M. & Mizumori, S. J. Y. Selective Functional Interaction Between the Lateral Habenula and Hippocampus During Different Tests of Response Flexibility. *Front. Mol. Neurosci.* **12**, 245 (2019).

143. Nielson, H. C. & McIver, A. H. Cold stress and habenular lesion effects on rat behaviors. *Journal of Applied Physiology* **21**, 655–660 (1966).

144. Shabel, S. J., Proulx, C. D., Trias, A., Murphy, R. T. & Malinow, R. Input to the lateral habenula from the basal ganglia is excitatory, aversive, and suppressed by serotonin. *Neuron* **74**, 475–81 (2012).
